# Optimal control of the coronavirus pandemic with both pharmaceutical and non-pharmaceutical interventions

**DOI:** 10.1007/s40435-022-01112-2

**Published:** 2023-02-01

**Authors:** Segun I. Oke, Matthew I. Ekum, Olalekan J. Akintande, Michael O. Adeniyi, Tayo A. Adekiya, Ojodomo J. Achadu, Maba B. Matadi, Olaniyi S. Iyiola, Sulyman O. Salawu

**Affiliations:** 1 Department of Mathematics, Ohio University, Athens, OH 45701-2979 USA; 2 Department of Mathematical Sciences, Lagos State University of Science and Technology, Ikorodu, Lagos Nigeria; 3 Computational Statistics Unit, Department of Statistics, University of Ibadan, Ibadan, Nigeria; 4 Department of Mathematical Sciences, University of Zululand, Richards Bay, South Africa; 5 Department of Pharmaceutical Sciences, College of Pharmacy, Howard University, Washington, DC USA; 6 Department of Science, School of Health and Life Sciences, Teesside University, Middlesbrough, TS1 3BA UK; 7 Department of Mathematics, Clarkson University, Potsdam, NY USA; 8 Department of Mathematics, Bowen University, Iwo, Nigeria

**Keywords:** COVID-19, Physical distancing, Time series data, Quarantine, Stability analysis, Data visualization

## Abstract

Coronaviruses are types of viruses that are widely spread in humans, birds, and other mammals, leading to hepatic, respiratory, neurologic, and enteric diseases. The disease is presently a pandemic with great medical, economical, and political impacts, and it is mostly spread through physical contact. To extinct the virus, keeping physical distance and taking vaccine are key. In this study, a dynamical transmission compartment model for coronavirus (COVID-19) is designed and rigorously analyzed using Routh–Hurwitz condition for the stability analysis. A global dynamics of mathematical formulation was investigated with the help of a constructed Lyapunov function. We further examined parameter sensitivities (local and global) to identify terms with greater impact or influence on the dynamics of the disease. Our approach is data driven to test the efficacy of the proposed model. The formulation was incorporated with available confirmed cases from January 22, 2020, to December 20, 2021, and parameterized using real-time series data that were collected on a daily basis for the first 705 days for fourteen countries, out of which the model was simulated using four selected countries: USA, Italy, South Africa, and Nigeria. A least square technique was adopted for the estimation of parameters. The simulated solutions of the model were analyzed using MAPLE-18 with Runge–Kutta–Felberg method (RKF45 solver). The model entrenched parameters analysis revealed that there are both disease-free and endemic equilibrium points. The solutions depicted that the free equilibrium point for COVID-19 is asymptotic locally stable, when the epidemiological reproduction number condition $(R_{0}<1)$. The simulation results unveiled that the pandemic can be controlled if other control measures, such as face mask wearing in public areas and washing of hands, are combined with high level of compliance to physical distancing. Furthermore, an autonomous derivative equation for the five-dimensional deterministic was done with two control terms and constant rates for the pharmaceutical and non-pharmaceutical strategies. The Lagrangian and Hamilton were formulated to study the model optimal control existence, using Pontryagin’s Maximum Principle describing the optimal control terms. The designed objective functional reduced the intervention costs and infections. We concluded that the COVID-19 curve can be flattened through strict compliance to both pharmaceutical and non-pharmaceutical strategies. The more the compliance level to physical distance and taking of vaccine, the earlier the curve is flattened and the earlier the economy will be bounce-back.

## Introduction

A group of patients from Wuhan, China republic, with unknown cause of pneumonia were in recent year observed [1, 2]. A previous novel betacoronavirus, otherwise known as 2019-ncov, SAR-COV-2, or COVID-19, was discovered in samples from this group of patients through the use of unbiased sequencing [1, 2]. A few weeks after, the virus was reported to have had spread to all round the world, making it a pandemic. Currently, over 2,034,000 established cases and death of about 134,500 documented in 213 nations around the globe, with report of new cases each day (WHO COVID-19 dashboard, [3]). Viruses are smallest infectious disease agents, apart from prions—agents of diverse neurodegenerative diseases. Viruses are made up of small genome, contained a single nucleic acid type (either DNA or RNA), which may be double stranded or single stranded in either case [4]. This genetic makeup is usually coated with a protein, and some viruses are further encased by a lipid envelope [5]. Viruses, unlike other infectious organisms, are the only group of organisms that cannot replicate outside of a host cell. In other words, viruses cannot reproduce and survive without a host cell. They lack ribosomes, which is required for the synthesis of proteins; instead, they use the ribosomes of their host’s cells to translate viral mRNA into viral proteins [5–7].

Viruses also lack the ability to produce or store its own energy in the form of ATP. Nevertheless, they make use of a host cell to secrete their energy and to perform other metabolic activities via the process known as self-replicating mechanism [4, 6]. Coronaviruses are types of viruses that are encased by RNA and are widely spread in humans, birds, and other mammals, leading to hepatic, respiratory, neurologic, and enteric diseases [1, 2]. There are six known coronavirus species that are disease-causing agents in man, namely OC43, 229E, HKU1, NL63, MERS-CoV, and SARS-CoV, while OC43, 229E, HKU1, NL63 are widespread and responsible for common cold or severe pneumonia symptoms in immunocompetent persons [1, 8], MERS-CoV and SARS-CoV are responsible for acute severe respiratory syndrome, middle-east respiratory disorder and coronavirus, respectively, their origin is zoonotic, and they have been associated with fatal illnesses[1]. With the emergence of 2019-nCoV, SAR-CoV, or COVID-19, a new strain of coronavirus undergoes self-replication machinery by using the protease secreted from the host cell to form a viral replication complex.

Some of the symptoms resulting from the infection of this virus include diarrhea, sore throat, tiredness, dry cough, fever, runny nose, nasal congestion, pain, aches, loss taste or smell sense, and even death, depending on the patients [1, 2]. Although the transmission of the virus is not well understood,, several studies have reported some possible ways of transmission, such as importation and human-to-human transmission in Vietnam [9] and person-to-person transmission [10]. Other studies in which the transmission of COVID-19 has been investigated include the transmission of the infection from an asymptomatic contact [11], prediction of international and domestic blowout of 2019-nCoV outbreak from Wuhan [12, 13], and early dynamic spread of pneumonia coronavirus disease in Wuhan, China [14]. A fractional approach to modeling this complex system was proposed in [15] where contact tracing, among others, was a key factor in mitigating the spread of the virus. Based on clinical expertise and currently available information, people of any age with underlying medical conditions (asthmatic, HIV-AIDS, tuberculosis patients, cancer among other serious illnesses) and the elderly may be at serious hazard of COVID-19 (CDC, 2021).

Even with the identification of specific treatments and vaccines to contain, there is the exponential increase in this disease; hence, other preventive measures, such as self-isolation, quarantine, and immune booster, are to be put in place. Quarantine plays an essential role in disease control mechanism and also acts as a preventive measure during imperfect vaccines or treatments. This implies that individuals who are exposed and infected are isolated in a secure area, in order to lessen the dispersion of the disease. This approach has been recently reported by several studies [16–20]. It is a common knowledge that the rapid, efficient, and ultrasensitive discover of the SARS-CoV-2 is critical for the prevention and/or control of the outbreak [21, 22]. Hence, the rush for information on surveillance and diagnostic machineries for SARS-CoV-2 has been triggered globally. In order to effectively deal with the outbreak, timely diagnostics are critical. With proper diagnostics in place, healthcare workers can be well informed on where and how to channel both resources and efforts to treat/isolate patients.

Thus, all the technologies needed to rapidly detect COVID-19 (SARS-CoV-2) are highly invaluable to the frontline policy makers and health care workers who strive in the joint efforts to ameliorate the scourge of the disease and/or bring to a halt its spread. Since the onset of the COVID-19 in the China Province of Hubei, different approaches have been adopted, globally, for its detection. Among the reported methods are the microscopy electron transmission, employed to detect the SARS-CoV-2 morphology[2]; genome sequencing, adopted to establish the virus identity [23, 24]; and data sequence, for the eventual detection [25]. The unfolding of new variants of acutely severe respiratory disorder coronavirus type-2 (SAR-CoV-2), particularly the clinical concern cases, has impacted the spreadability and infectivity of the virus, as well as the diagnostic measures and efficacy of vaccines employed as mitigation strategies, though most of the SARS-CoV-2 mutated variants are either quite harmful or neutral, clearing up rapidly. It has also been observed that some of the variants severely affected exposed humans while possibly altering infection rates and/or symptoms severity along the way, thus perturbing the immune system.

In late 2020, SARS-CoV-2, evolved from the previous year, was marked by the advent of new variants that were characterized with viral traits changes such as antigenicity and transmissibility, mainly due to the compromise in human immunological system upon infection by the new viral pathogen. As a “variant of interest,” the Delta variant was first discovered in India at the end of 2020. By mid-2021, Delta variant infections were recorded in over 163 countries. The World Health Organization (WHO) then declared the Delta strain as the most widespread and dominant strain in the world, thereby branding it as a “variant of concern (VOC)”[26]. According to clinical data and epidemiological surveys, the Delta VOC SARS-CoV-2 is potentially highly transmissible at 40–60 percent (%) rate than the Alpha or Beta strains, with a substantial risk of causing illnesses that is largely responsible for the high rate of hospitalization [27, 28]. Thus, the Delta VOC mostly endangers those that are unvaccinated, with fewer casualties reported in vaccinated people [26, 29, 30]. Despite the fact that the Delta variant emerged during the second wave of Indian’s SARS-CoV-2 invasion, this strain dominated internationally with new clinical data knowledge still emerging from around the world.

Toward the end of 2021, WHO announced that another mutant strain of SARS-CoV-2, the B.1.1.529 variant, had been discovered and quickly named it Omicron. This variant exhibited several mutations that could affect its properties [26, 30, 31]. Although knowledge of the extent of the transmissibility and infectivity of Omicron variant is slowly emerging, it is already considered a VOC due to the multiple mutation sites on the spike protein. This is likely to affect how quickly the Omicron is dispersed or the harshness of the caused illness. The variation in the spike protein is characterized by more than 30 mutations, of which is happen at the recombinant binding site, as well as 1 minor insertion and 3 minor deletions [26, 29, 31]. The Omicron strain was reportedly discovered in South Africa and Botswana on November 2021, but before the end of 2021 November, travel-related incidents have also been documented in Israel, Hong Kong, Belgium, the UK, USA, and Netherlands [26, 29, 32]. The Omicron variant appears to be a quite diverse variant and thus raised concerns likely higher transmissibility and vaccine resistance, coupled with a high tendency of re-infection.

Overall, the number of countries that have reported Omicron VOC SARS-CoV-2 disease spread continues to increase, with highest daily average cases of 799,000 (Omicron) and 164,000 (Delta) confirmed in over 30 countries at the start of 2022 [32, 33]. However, it remains ambiguous whether or not the existing Omicron SARS-CoV-2 variant could deleteriously cause clinical crises and hospitalizations compared to the dreaded Delta strain. It is hoped that various studies underway are able to establish a solid understanding of the clinical and epidemiological effects of Omicron and Delta variants compared to the Wuhan-Hu-1 strain that emerged in 2019. Both variants are spread from person to person through physical contact. Thus, maintaining physical distance and taking the COVID-19 vaccine can help to reduce the spread and its effects on persons.

Thus, this present study focused on the influence of immigration, person-to-person transmission, quarantine individuals who has been tested with no clinical symptoms, social distancing of susceptible individual, vaccinated individuals, and effect of loss of immunity, using mathematical and statistical approach. This approach is an important tool in getting insights into transmission of diseases, and it is applicable in making decisions in regard to intervention machineries for infectious disease mitigation [34].

### Related works

Recently,[35] used dynamic model approach to analyze the influence of isolation and quarantine in dynamics of transmission of the MERS-CoV in relation to latent immigrants. The authors revealed that instant isolation, close monitoring of quarantining, and contacts of individual asymptomatic immigrants can be helpful in MERS-CoV control. The numerical simulations in their study further revealed that the combination of a great reduction in the number of immigration, as preventive measures, can help to contain MERS-CoV. Similarly, Dighe and co-workers investigated the transmission of MERS-CoV in Camelus dromedaries, dromedary camels. It was shown that MERS-CoV in camels have moderate transmissibility. It was also revealed by a metapopulation model of MERS-CoV transmission that camel populations in the Arabian Peninsula and Africa have the long-term persistence of MERS-CoV, and this can be helpful in the simulation of camel vaccination strategies. Khan and Atangana[13] traced COVID-19 disease to the seafood market when the bats and other hosts are unknown, and this was done to give insight to the dynamic of the novel coronavirus. The fractional model revealed the stability of the disease to be asymptomatic when $R_{0} < 1$, and the statistical data showed the basic reproduction to be $R_{0} = 2.4829$ in all. The results generated in their study can be helpful in minimizing the infection.

Zhao and co-workers [36] used a data-driven and mathematical approach to analyze the early stage outbreak of the COVID-19 by estimating the basic reproduction number in China from 2019 to 2020. The findings revealed $R_{0}$ to be greater than 1, which is a possible indication of the virus outbreak as predicted by their findings. Zhang et al. [37] also employed a data-derived analysis to investigate the reproductive number of COVID-19 in the Diamond Princess cruise ship and predicted the early stage outbreak size. Social distancing (SD) practices can be described as the reduction in the rates of contact between vulnerable and individuals that are already infected with a disease and who may transmit the disease [38]. SD is a change in behavior aimed at reducing the severity of an epidemic, or in this case, pandemic. Buttressing this, Valdez et al. [39] add that SD is a recurrent social distancing strategy in which healthy persons are encouraged to prevent contact with their neighbors for a period of time. However, Reluga [38] observed that the advantages of social distance are determined by the extent to which individuals comply, as most people are sometimes reluctant to pay the costs, which in turns reduces its effectiveness as a control measure.

In their study, Valdez et al. [39] successfully used percolation tools to show that SD is a strategy that can put a stop to the spread of epidemics [39]. In the same vein, Shim [40] adopted vaccination and social distancing optimal strategies and proved that SD is effective against the spread of seasonal influenza [40]. The optimization of a dynamical objective of a nonlinear system for the measures of time varying control described control theory offers [41]. This approaches have been most employed in the study of various diseases transmission dynamics such as [40, 41]. The latter investigations depict that the non-pharmaceutical interventions level depends on the entrenched terms, and its application is required for a long duration and high level without vaccine. Here, an optimal control technique is implemented to examine the non-pharmaceutical interventions optimal strategies on the COVID-19 control: case study of USA. The weighing of the relative cost of COVID-19 mortality and control is optimized in strategy, and determine a technique, minimize, and control combined cost. Other analysis of COVID-19 optimal control begins to emerge [42–44], though these are given less attention on a geographic particular location.

Abbas et al. [45] presented a fractional model and performed some simulations, which validated their analytical findings using a discritization method. [46] presented and discussed some approaches used in modeling and surveillance of infectious diseases dynamics, by considering asymptomatic and symptomatic stages of infections. After they had highlighted the conceptual ideas and the mathematical tools needed for the modeling, they computed basic reproduction number and investigated the qualitative behaviors of the disease via simulation study. [47] presented the population migration model for *n*-cities and applied the model for migration between two and three cities. They computed their reproduction number, analyzed the effect of the migration rate, and simulated a protocol of repeated lock-downs that limits the resurgence of infections, and observed a damped oscillatory behavior with multi-modal for some periods under reveal. [48] proposed a SEIQR (Susceptible–Exposed–Infected–Quarantined–Recovered) mathematical model and its control measurement. They computed the basic reproduction number for Russia and India and concluded that future predictions from mathematical model and the LSTM based model are compared to generate reliable result.

In this study, reported cases are considered for the first 705 days, that is from January 22, 2020, to December 20, 2021. The study collected data on fourteen countries, out of which the model was simulated using four selected countries: USA, Italy, South Africa, and Nigeria. The first 12 countries with the highest reported cases were selected, while South Africa and Nigeria were selected based on authors interest. The four countries are selected based on severity of disease and interest. USA and Italy were selected because the had the highest and second highest recorded deaths, while South Africa and Nigeria were selected based on interest. Thus, the study arrangement is as follows: In Sect. 2, we formulated a dynamical COVID-19 mathematical formulation to parameterize the model and presented the local stability analysis and global stability, using Lyapunov function, while analysis of both local and global sensitivities was carried out using elasticity index and partial rank correlation coefficient (PRCC), and in Sect. 3, we have the model numerical simulations and parametric estimation. We further explored some fundamental properties of the model and discussions in Sect. 4, while the findings and conclusion are presented in Sect. 5.

## The description of mathematical model

The entire population of people is depicted by $N_{h}$, which is classified into five subgroups such as $S_{h}$, $Q_{h}$, $I_{h}$, $R_{h}$, and *D*, respectively, the Susceptible, Quarantines, Infected (symptomatic), Recovered, and Death. The human susceptible population is recruited through the birth rate (at a constant rate) $\Pi$ and immigration via (Air, Road & Sea) borders (at the rate $\alpha ~ m$), where $0 \le m \le 1$ denotes the migrants inflow fraction into the susceptible countries, and individual recovery from the quarantine at $\delta _{0} (1-p_{1})$ rate, who has been tested without COVID-19 clinical symptoms, where $0< p_{1} < 1$ defined the probability of the infected individual transmits the disease to susceptible individual. Hence, the population is decreasing by an acquired COVID-19 through proper infectious human contact (at $\lambda$ rate) where $\lambda = \frac{p_{1} \beta I_{h}}{N_{h}}$ and $\beta$ denotes rate contact sufficient (i.e., enough to cause COVID-19 infection). The birth rate of the susceptible is given by $\Pi _{h}$ while the rate of natural death in each class is given by $\mu _{h}$. Here, the susceptible changing rate is expressed as:

2.1 $$\begin{aligned} S'_{h}(t){} & {} = \Pi + \alpha m - \left( \lambda + \mu + \tau \right) S_{h}(t)\nonumber \\{} & {} \quad + \delta _{0}(1 - p_{1})Q_{h}(t) + \delta _{1}R_{h}(t), \end{aligned}$$

The susceptible population $S_{h}$ will be increase or generated via birth and rate of immigration of $\alpha m$ & $\delta _{0} (1-p_{1})$ and infection (at $\lambda$ rate) and is reduced by $\tau$ fraction of people who practice social distancing, also reduced by people that developed of clinical symptoms.

2.2 $$\begin{aligned} Q'_{h}(t)= \lambda S_{h}(t) - \left( \mu + \theta + \delta _{0}(1 - p_{1}) \right) Q_{h}(t), \end{aligned}$$

The quarantine individual population rises following the quarantine of persons in the susceptible class (at $\lambda$ rate). This population reduced by recovery $\delta _{0}$, progression rates to infected class $\theta$, and natural death.

2.3 $$\begin{aligned} I'_{h}(t)= \theta Q_{h}(t) - \left( \mu + \sigma + \gamma \right) I_{h}(t) \end{aligned}$$

The infected compartment with clinical symptoms for COVID-19 in $I_{h}$ class rises going the development of clinical symptoms in quarantine (at $\theta$ rate) class. This class reduced by COVID-19 induced death (at a rate $\sigma$), recovery (at a rate $\gamma$), and natural death.

2.4 $$\begin{aligned} R'_{h}(t)=\gamma I_{h}(t) + \tau S_{h}(t) - (\mu + \delta _{1})R_{h}(t) \end{aligned}$$

The recovery class is generated by the recovered population from infected class (Tables 1 and 2). In with the rate at $\gamma$ and fraction of susceptible population who practice social distancing during the pandemic (at $\tau$ rate) and decrease as a result of natural death.

2.5 $$\begin{aligned} D'_{h}(t)= \sigma I_{h}(t) \end{aligned}$$

The death population is generated by the COVID-19-induced death with the rate at $\sigma$

2.6 $$\begin{aligned} {\left\{ \begin{array}{ll} S_{h}'(t)= \Pi + \alpha m - \left( \lambda + \mu + \tau \right) S_{h}(t) \\ + \delta _{0}(1 - p_{1})Q_{h}(t) + \delta _{1}R_{h}(t),\\ Q_{h}'(t)= \lambda S_{h}(t) - \left( \mu + \theta + \delta _{0}(1 - p_{1}) \right) Q_{h}(t) ,\\ I_{h}'(t)= \theta Q_{h}(t) - \left( \mu + \sigma + \gamma \right) I_{h}(t) ,\\ R_{h}'(t)=\gamma I_{h}(t) + \tau S_{h}(t) - (\mu + \delta _{1})R_{h}(t)\\ D_{h}'(t)= \sigma I_{h}(t), \end{array}\right. } \end{aligned}$$

where $$\lambda = \frac{p_{1}\beta I_{h}}{N_{h}}$$, $$N_{h}= (S_{h} + Q_{h}+ I_{h} + R_{h} + D_{h}) $$

**Table 1:** Description of entrenched terms in the model (2.6)

Variable	Symbol
Susceptible	$S_{h}(t)$
Quarantine	$Q_{h}(t)$
Infected	$I_{h}(t)$
Recovery	$R_{h}(t)$
Death	$D_{h}(t)$

**Table 2:** Description of parameters, values, and their units

Parameter	Symbol	Value	95% CI	Unit	Refs.
Recruitment rate via birth	$\Pi$	0.01218	(0.0063, 0.0181)	day$^{-1}$	[35]
Human immigration recruitment rate	$\alpha$	0.00401	(0.0000, 0.0085)	day$^{-1}$	[35]
Immigrant fraction	*m*	0.39152	(0.0000, 0.9886)	day$^{-1}$	Estimated
Probability that a susceptible will get infected	$p_{1}$	0.71981	(0.4085, 1.0000)	day$^{-1}$	Estimated
Natural death for all compartment	$\mu$	0.01277	(0.0107, 0.0148)	day$^{-1}$	Estimated
Recovery of quarantined individuals who has been tested of Covid-19 without clinical symptoms	$\delta _{0}$	0.01130	(0.0000, 0.1850)	day$^{-1}$	Fitted
Progression rates to infected class	$\theta$	0.48022	(0.4556, 0.5049)	day$^{-1}$	Fitted
Recovery rates for infectious individual to recovery class	$\gamma$	0.28185	(0.0000, 0.6099)	day$^{-1}$	Estimated
Rate of compliance to social distance practice	$\tau$	0.00058	(0.0000, 0.0015)	day$^{-1}$	Fitted
Rate at which recovered persons losses immunity and progress to Susceptible class	$\delta _{1}$	0.0001	(0.0000, 0.0003)	day$^{-1}$	Estimated
Covid-19-induced death rate	$\sigma$	0.20704	(0.0000, 0.4420)	day$^{-1}$	Estimated
Effective contact rate	$\beta$	0.98513	(0.4000, 0.9950)	day$^{-1}$	Estimated

## The dynamic behaviors of the proposed COVID-19 model

The dynamic behaviors of the proposed COVID-19 model are the focus of this section. Several qualitative analyses are carried to examine how well the model formulated is able to capture the dynamic nature of the deadliest infectious disease called COVID-19. Rigorous stability analysis results are presented including the solutions boundedness and positivity and the results of the steady-state model. For simplicity, we denote the following parameters as:

$$\begin{aligned} \Psi _{1}= & {} \delta _{0}(1 - p_1)\ge 0 \\ \Psi _{2}= & {} \theta +\mu + \Psi _{1} \ge 0 \\ \Psi _{3}= & {} \gamma +\sigma + \mu \ge 0 \\ \Psi _{4}= & {} \tau + \delta _{1} + \mu \ge 0 \\ \Psi _{5}= & {} \beta p_1(\delta _{1} + \mu ) \ge 0 \\ \Psi _{6}= & {} \delta _{1} + \mu \ge 0 \\ \end{aligned}$$

### Positivity and boundedness of solutions

Here, the system (2.6) is established to be epidemiological realistic and well posed, when all terms in system (2.6) at all times *t* are non-negative. Therefore, Lemma 1 is defined to establish this.

#### Lemma 1

The solutions $S_{h}(t), Q_{h}(t),I_{h}(t),R_{h}(t)$, and $D_{h}$ (*t*) of the system (2.6) subject to initial conditions $S_{h}(0)>0, Q_{h}(0)\ge 0,I_{h}(0)\ge 0,R_{h}(0)\ge 0$ and $D_{h}(0)\ge 0$ are positive for all $t>0$.

#### Proof

From the system (2.6), we have

$$\begin{aligned} \frac{\mathrm{{d}}S_{h}}{\mathrm{{d}}t} \vert _{S_{h}=0}= & {} \Pi +\alpha m + \Psi _{1}Q_{h} + \delta _{1}R_{h}\ge 0\\ \frac{\mathrm{{d}}Q_{h}}{\mathrm{{d}}t} \vert _{Q_{h}=0}= & {} \lambda S_{h}\ge 0\\ \frac{\mathrm{{d}}I}{\mathrm{{d}}t} \vert _{I_{h}=0}= & {} \theta Q_{h}\ge 0\\ \frac{\mathrm{{d}}R_{h}}{\mathrm{{d}}t} \vert _{R_{h}=0}= & {} \gamma I_{h} + \tau S_{h}\ge 0\\ \frac{\mathrm{{d}}D_{h}}{\mathrm{{d}}t} \vert _{D_{h}=0}= & {} \sigma I_{h}\ge 0. \end{aligned}$$

Thus, the rate defined above is non-negative on bounded plane $\Re ^{5}_+$; hence, it shows that the region is attracting and positively invariant. In system (2.6), the attractive region is expressed as

3.1 $$\begin{aligned} \Omega= & {} \left\{ (S_{h}, Q_{h}, I_{h}, R_{h}, D_{h})\in \Re ^{5}_{+}: S_{h}+Q_{h}\right. \nonumber \\{} & {} \left. +I_{h}+R_{h} \le \frac{\Pi + \alpha m}{\mu } \right\} \end{aligned}$$

Therefore, the sufficient system of dynamic (2.6) in *Omega* is considered and it attracts all initiating solutions in the interior non-negative invariant. $\square$

We next prepare ground for stability analysis for the proposed model (2.6) by considering the basic reproduction number is denoted by $R_{0}$ and the COVID-19-free equilibrium point. The COVID-19 free equilibrium point for the system (2.6) is

$$\begin{aligned} E_{0}= & {} (S^{*}_{h}, Q^{*}_{h}, I^{*}_{h}, R^{*}_{h}, D^{*}_{h})\\= & {} \left( {\frac{ \Psi _{6} \left( \alpha \,q+\Pi \right) }{\mu \, \Psi _{4}}}, 0, 0, {\frac{\tau \, \left( \alpha \,q+\Pi \right) }{\mu \, \Psi _{4}}}, 0 \right) \end{aligned}$$

The calculation of basic reproduction number is done. The Local stability analysis of the COVID-19 free equilibrium (CFE) is carried out by adopting the next-generation operator technique [49, 50] where necessary computations of the matrices *F* and *V* are shown by:

$$\begin{aligned} F= & {} \begin{pmatrix} \begin{array}{cc} 0&{}{\frac{\Psi _{5} }{\Psi _{4}}}\\ 0&{}0\end{array} \end{pmatrix}\\ V= & {} \begin{pmatrix} \begin{array}{cc} \Psi _{2} &{}0\\ -\theta &{} \Psi _{3} \end{array} \end{pmatrix}\\ V^{-1}= & {} \begin{pmatrix} \begin{array}{cc} \frac{1}{\Psi _{2}} &{}0\\ {\frac{\theta }{ \Psi _{2}\Psi _{3} }}&{} \frac{1}{\Psi _{3}} \end{array} \end{pmatrix}. \end{aligned}$$

The $\gamma (FV^{-1})$ of the spectral radius is needed basic reproduction number of the system (2.6), expressed as

$$\begin{aligned} R_{0}= \frac{\theta \Psi _{5}}{\Psi _{2}\Psi _{3}\Psi _{4}} \end{aligned}$$

### COVID-19 free equilibrium local stability, $E_{0}$

The investigation of local stability of $E_{0}$ is carried out by adopting Theorem:

#### Theorem 1

The free equilibrium for COVID-19 $E_{0}$ is asymptotically and locally stable for the system (2.6) when $R_{0}<1$ or else unstable.

#### Proof

Take the Jacobian matrix of Eq. (2.6) and evaluate at $(E_{0})$

3.2 $$\begin{aligned} J(E_0) = \begin{pmatrix} \begin{array}{cccc} -\mu -\tau &{} \Psi _{1} &{}-{\frac{\Psi _{5}}{\Psi _{4}}} &{} \delta _{{1}}\\ 0 &{} -\Psi _{2} &{} {\frac{\Psi _{5}}{\Psi _{4}}}&{}0\\ 0&{}\theta &{} -\Psi _{3} &{}0 \\ \tau &{}0&{}\gamma &{} -\Psi _{6} \end{array} \end{pmatrix}. \end{aligned}$$

According to Routh–Hurwitz condition,

$$\begin{aligned} (i) \quad \mathrm{{Trace}}(E_{0})<0 \quad (ii)\quad \mathrm{{Determinant}} (E_{0})>0 \end{aligned}$$

Clearly,

$$\begin{aligned}&\mathrm{{Tr}}(E_{0})=-(\mu + \tau + \Psi _{2} + \Psi _{3} + \Psi _{6})<0\\&\begin{aligned}&\mathrm{{Det}}(E_{0}) \\&\quad = -{\frac{\left[ \beta \,\mu \,\theta \,p_{1} + \beta \,\theta \,\delta _{1}p_{{1}}-\Psi _{2}\Psi _{3}\Psi _{4}\right] \left[ (\mu +\tau )\,\Psi _{6}-\tau \,\delta _{1}\right] }{\Psi _{4}}}\\&\quad =\frac{-1}{\Psi _{4}}((\mu + \delta _{1})(\mu + \tau )-\tau \delta _{{1}}) \\&\qquad \left( (\delta _{{1}}+\mu )\beta p_{1} \theta - \Psi _{2}\Psi _{3}\Psi _{4}\right) \\&\quad = -\mu \Psi _{2}\Psi _{3}\Psi _{4}\Bigg (\frac{(\delta _{{1}}+\mu )\beta p_{1} \theta }{\Psi _{2}\Psi _{3}\Psi _{4}} -1\Bigg )\\\\&\quad =-\mu \Psi _{2}\Psi _{3}\Psi _{4}(R_{0}-1)>0 \quad if\quad R_{0}<1. \end{aligned} \end{aligned}$$

This implies that the eigenvalues of the equation (2.8) are negative and real when $R_{0}<1$. Hence, CFE $E_{0}$ is asymptotically and locally stable and unstable when $R_{0}>1$. $\square$

### Global asymptotically stable of CFE, $E_{0}$

Given that the stability of CFE, $E_{0}$ has nothing yo do with the initial population size while the local stability of $E_{0}$ did not hold for the dominating conditions; therefore, global asymptotic stability (GAS) is considered. To carry it out, a Lyapunov function is established. This method has been used by several authors [41] to prove the global stability of epidemiological steady states. Assume a Lyapunov function denoted as:

3.3 $$\begin{aligned} L(Q,I)=\theta Q_{h} + \Psi _2 I_{h} \end{aligned}$$

Obtaining the derivative of equation (2.10) together with the solutions of Eq. (2.6) gives

$$\begin{aligned} L'(Q,I)&= \theta Q^{'}_{h} + \Psi _2 I^{'}_{h} \\&= \theta \left[ \lambda S_{h} - \Psi _{2} Q_{h}\right] + \Psi _2 \left[ \theta Q_{h} - \Psi _{3}I_{h}\right] \\&=\theta \lambda S_{h} - \theta \Psi _{2} Q_{h} + \Psi _2 \theta Q_{h} - \Psi _2 \Psi _3 I_{h}\\&= \left[ \theta \frac{\beta p_{1} I_{h}S_{h} }{N_{h}} - \Psi _2 \Psi _3 I_{h}\right] \\&= \left[ \theta \frac{\beta p_{1} S_{h} }{N_{h}} - \Psi _2 \Psi _{3} \right] I_{h}\\ \end{aligned}$$

Hence, at CFE, $E_{0}$, we have $\displaystyle N_{h} = S_{h} + R_{h}$,   $\displaystyle N_{h}=\frac{\pi + \alpha m}{\mu }$, $\displaystyle \frac{S_{h}}{N_{h}} = \frac{(\mu + \delta _{1})}{(\mu + \tau + \delta _{1})} = \frac{(\mu + \delta _{1})}{\Psi _4}$, and $m_{1}= \theta$.

so that

$$\begin{aligned} L^{'}(Q,I)&= \left[ \theta \frac{(\mu + \delta _{1})}{\Psi _4}\beta p_{1} - \Psi _2 \Psi _3 \right] I_{h} \\&=\left[ \frac{(\mu + \delta _{1})}{\Psi _4}\beta p_{1} \theta - \Psi _2 \Psi _3 \right] I_{h}\\&= \Psi _2 \Psi _3\left[ \frac{\theta \Psi _5}{\Psi _2 \Psi _3\Psi _4} -1 \right] I_{h}\\ \end{aligned}$$

Therefore,

3.4 $$\begin{aligned} L^{'}(Q,I)=\Psi _2 \Psi _3\left[ R_{0}-1 \right] I_{h}\le 0 \quad if \quad R_{0}\le 1 \end{aligned}$$

Thus, the CFE $E_{0}$ is global asymptotically stable when $R_{0}\le 1$ or else unstable. This could be summarized as:

#### Theorem 2

The CFE $E_{0}$ is globally asymptotically stable if $R_{0}\le 1$ or else unstable.

### The model (2.6) local and global sensitivity analysis

In this section, parameter sensitivities are investigated. Taking from [51, 52], the local sensitivity of model parameters is calculated by elasticity index. The local sensitivity analysis is performed on the basic reproduction number $R_{0}$. The parameter $R_{0}$ is employed to check the effect of COVID-19 pandemic in all selected countries. This above technique is applied to determine the quantity parameter changes such as $\tau$, in respect to rate of change in quantity $R(\tau )$. The elasticity index or normalized sensitivity index of $R(\tau )$ in respect to $\tau$ is given below:

3.5 $$\begin{aligned} \gamma ^{R_{0}}_{\tau } =\frac{\tau }{R_{0}}\times \frac{\partial R_{0}}{\partial \tau } \end{aligned}$$

**Table 3:** $R_{0}$ sensitivity index in respect to model (2.6) parameters

Parameter	Sensitivity index
$\beta$	+1.00000
$\theta$	+1.00000
$P_{1}$	+1.03648
$\delta _{1}$	+0.00034
$\mu$	$-$0.03994
$\sigma$	$-$1.34124
$\tau$	$-$0.04312
$\delta _{0}$	$-$0.01420
$\gamma$	$-$0.56183

Table 3 shows that the probability that a susceptible person get infected $p_{1}$ has the first highest sensitivity index (S.I = 1.03648), followed by the active contact rate $\beta$ and infected progression rate $\theta$ which indicates that increasing (or decreasing) the parameters mentioned above, especially probability that a susceptible person get infected $p_{1}$ by 10%, will decrease or increase the $R_{0}$ by 10.37%. The second most sensitivity index (S.I = $-$ 1.34124) is the induced Covid-19 death, $\sigma$, followed by recovery rate of infected individual $\gamma$ and compliance rate to social distancing $\tau$. All these parameters can be increased (or decreased) $R_{0}$ by 10% that is (13.4%, 5.6% and 0.43%), respectively. Our analysis results show that the $p_{1}$ has the most sensitivity index; however, the probability of getting infected must be decreasing for $R_{0}$ to be reduce. Furthermore, the sensitivity index of compliance to social distancing need to be enforcing that is increasing from $(\tau = 0.43\%)$ to a reasonable percentage in all the selected countries for $R_{0}$ to be reduced and also for recovery rate to be increased. Thus, it is not medically reasonable to consider COVID-19-induced death $(\sigma )$ as a control strategy to reduce $R_{0}$. However, the most effective control strategy is to decreasing the effective contact rate $(\beta )$ and increasing the social distancing compliance enforcement in all the selected countries.

**Fig. 1:**
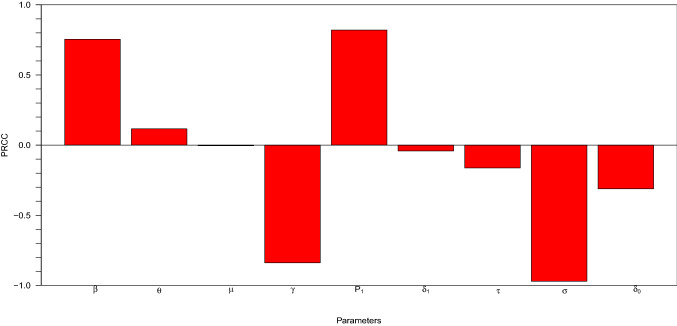
Partial rank correlation coefficient (PRCC) graphs plots for different terms in COVID-19 formulation (2.6), taking values of $R_{0}$ as the output function from Table 2

Variation in terms for the global sensitivity analysis for some estimated parameters by adopting baseline range values of Table 2. The partial rank correlation coefficient (PRCC) of the entrenched terms in the model is calculated and offered in Fig. 1. Parameters are sampled and substituted by Latin Hypercube Sampling (LHS) technique [41] (a valid statistical scheme for multidimensional distribution to obtaining parameter sample values). Total simulations of 1000 were carried out.

The baseline parameter values of Table 2 were changed in 25% range. Figure 1 offers PRCCs tornado plot versus the homogeneous parameter values in $R_{0}$ as the dependent baseline variable. The terms are momentously positively correlated with COVID-19 at $p < 0.05$ significance level being $\beta , \theta ~  \& ~ p_{1}$, while $\mu , \gamma , \delta _{1}, \tau , \sigma ~  \& ~ \delta _{0}$ are momentously negatively correlated. The infected class progression rate $\theta$, active contact rate $\beta$, infected susceptible probability reduce as social distancing compliance is effective and supported with hand washing, face mask wearing, and so on.

## Numerical simulations and data fitting

**Fig. 2:**
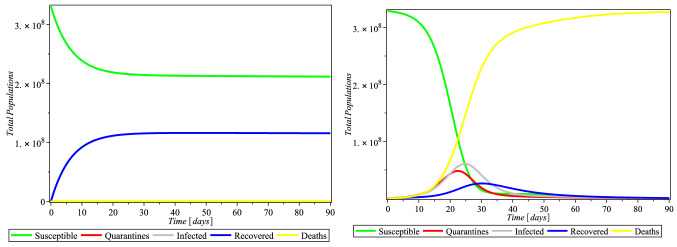
Simulations of the system (2.6) showing the free equilibrium point for COVID-19 and the epidemic equilibrium point

Here, we discuss the numerical solutions of Covid-19 formulation of (2.6). The computational procedure of Eq. (2.6) was using RKF45 Runge–Kutta–Fehlberg method via Maple solver, a member of explicit and implicit iteration schemes; these include the Euler’s method that used an approximate temporal discretization solution for ODEs of fourth order with fifth-order error estimator (Fig. 2).

**Fig. 3:**
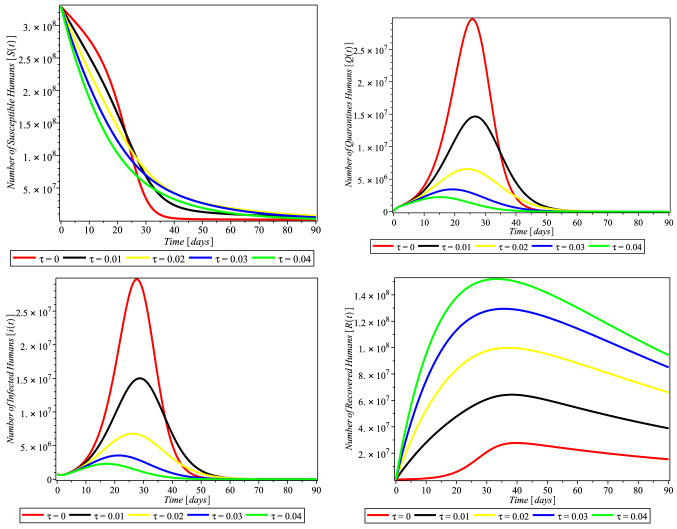
Compliance effect of social distancing parameter $\tau$ on the dynamics of COVID-19 model for USA. $\pi = 1.24X10^{-1}$, $\alpha = 3.8e-2$, $\beta = 0.9894$, $\gamma = 6.51X10^{-1}$, $m = 0.2599$, $\mu = 1.27X10^{-1}$, $\sigma = 0.3765$, $\theta = 0.4790$, $\delta _{0} = 8.3X10^{-5}$, $\delta _{1} = 2.440X10^{-4}$, $p_{1} = 0.9449$, $S(0) = 329247215$, $Q(0) = 72361$, $I(0) = 784326$, $R(0) = 72329$, $D(0) = 42094$

We consider four selected countries: USA, Italy, South Africa, & Nigeria, as case studies. The World Health Organization census data were adopted to define the total population for each country. It is assumed that people can move around within the continent. This, assumption is made based on the $\beta$, the sufficient rate of contact (i.e., COVID-19 infection rate). It is also assumed that the entire individuals in a country under consideration must be in any of the five partition (Susceptible, Quarantines, Infected (symptomatic), Recovered, and Death). This implies that an individual who is not dead and has never been infected with COVID-19 must either fall into the quarantined class or the susceptible class. In simple language, a person who is not COVID-19 infected is in the susceptible class.

**Fig. 4:**
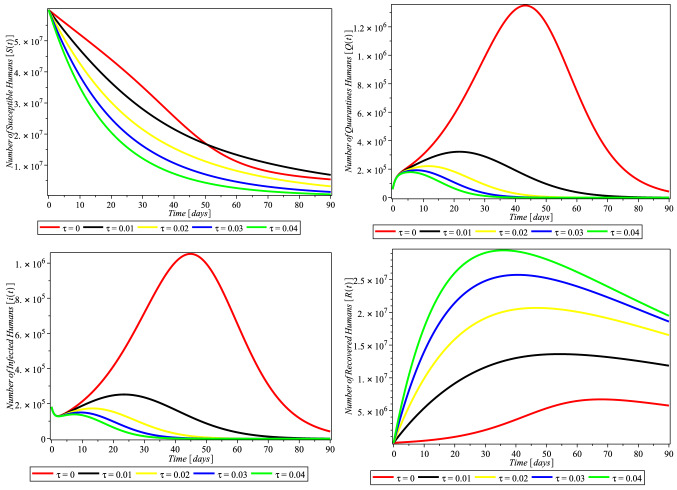
Simulations of the COVID-19 showing when the system is Covid-19 free equilibrium point and when it is epidemic equilibrium point for Italy. $\pi = 8.5X10^{-2}$, $\alpha = 3.6X10^{-2}$, $\beta = 0.9873$, $\gamma = 0.2199$, $m = 0.2754$, $\mu = 1.20X10^{-1}$, $\sigma = 0.3506$, $\theta = 0.4571$, $\delta _{0} = 1.3X10^{-3}$, $\delta _{1} = 1.69X10^{-3}$, $p_{1} = 0.7901$, $S(0) = 59956676$, $Q(0) = 59307$, $I(0) = 181228$, $R(0) = 59273$, $D(0) = 24114$

**Fig. 5:**
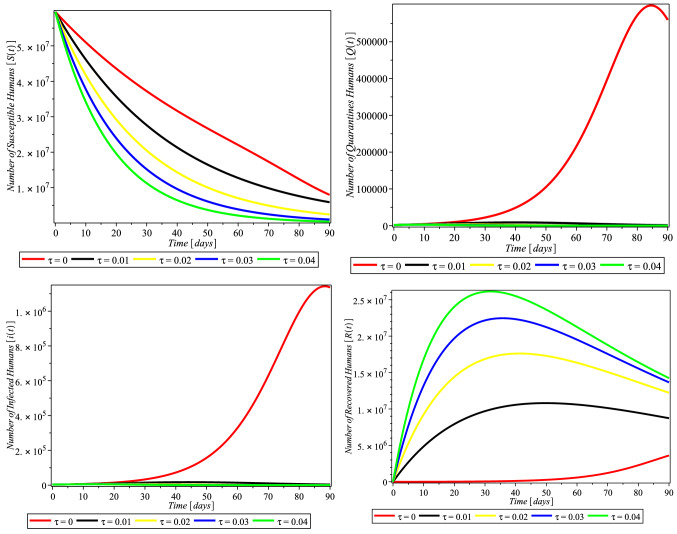
Compliance effect of Social distancing parameter $\tau$ on the dynamics of COVID-19 model for South Africa. $\pi = 2.10X10^{-1}, \alpha = 7.3X10^{-2}, \beta = 0.4772, \gamma =0 .1629, m =0 .1341, \mu = 1.56X10^{-1}, \sigma = 6.22X10^{-1}, \theta = 0.4746, \delta _{0} =0 .1364000, \delta _{1} = 4.1X10^{-6}, p_{1} = 0.8383,~ S(0) = 59605694, Q(0) = 1674, I(0) = 3300, R(0) = 1055, D(0) = 58$

**Fig. 6:**
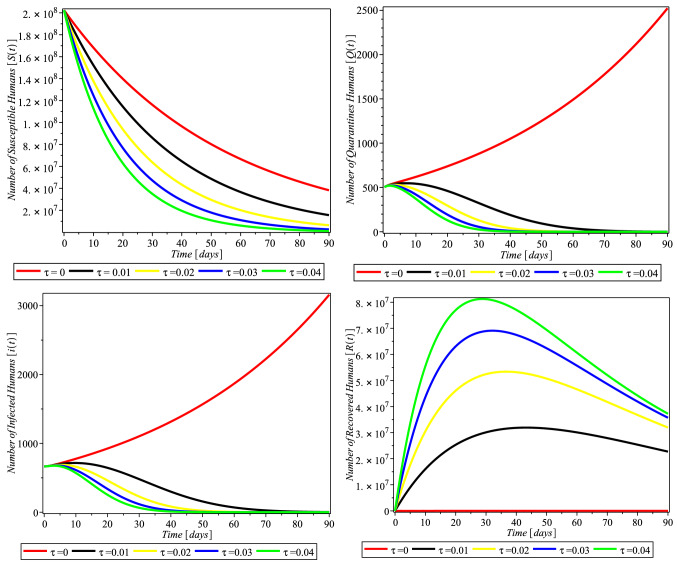
Compliance effect of social distancing parameter $\tau$ on the dynamics of COVID-19 model for Nigeria. $\pi = 3.80X10^{-1}, \alpha = 7.0X10^{-2}, \beta =0 .5294, \gamma = 0.2204, m = 0.1374, \mu = 1.85X10^{-1}, \sigma = 0.1077, \theta = 0.4559, \delta _{0} = 3.59000X10^{-1}, \delta _{1} = 9.0X10^{-7}, p_{1} =0 .7556, S(0) = 202376559, Q(0) = 505, I(0) = 665, R(0) = 188, D(0) = 22$

The rate of compliance to social distance practice ($\tau$) varies from county to country. Figure 3 shows that in USA, as the rate of compliance to social distance practice increased, susceptible individual numbers decreased faster, quarantine individual numbers decreased, infected individual numbers decreased significantly, and recovered individual numbers increased significantly as well. Figures 4 and 5 and 6 depict the same but clearer trend for Italy, South Africa, and Nigeria, respectively. This is inline with Centre for Disease Control (CDC) and World Health Organization (WHO) that compliance to social distance practice would decrease the virus spread. So, increasing the rate of compliance to social distance practice would significantly decrease the rate of COVID-19 infection (Fig. 6).

**Fig. 7:**
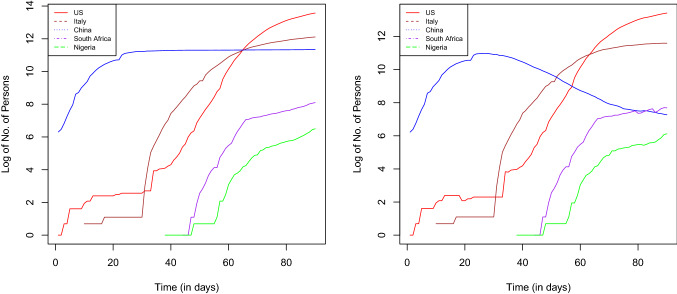
Time plot of cumulative COVID-19-confirmed cases (left) and active cases (right) of selected countries

Figure 7 (left) shows the COVID-19 cumulative confirmed laboratory cases of 5 chosen countries out of the 15 under study. As at April 20, 2020, only China curve was flattened as shown by the cumulative curve when compared to other selected countries. There is intersection among USA, Italy, and China at a point. At this point, USA and Italy surpassed China cases. The log is used rather than actual values so that all the curves are sighted. Figure 7 (right) shows the COVID-19 active cases of 5 selected countries out of the 15 under study. As at April 20, 2020, only China active cases curve is reducing and fast approaching zero. The active cases are those individuals that still have the disease after closed (recovery + death) cases have been subtracted from the cumulative infected cases.

**Fig. 8:**
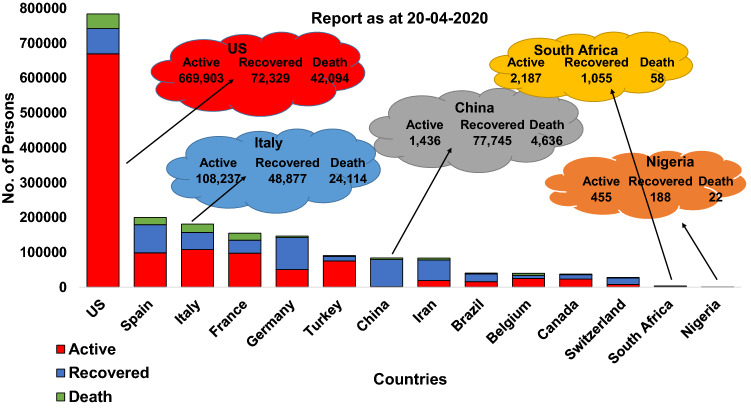
COVID1-19 active cases, recovered, and death of selected countries

Figure 8 shows a component bar chart of COVID-19 active cases, recovered, and death of 15 selected countries. At this period, China has the highest recovered individuals when compared with other countries. This is why the cumulative curve of infected cases of China is flattened.

**Fig. 9:**
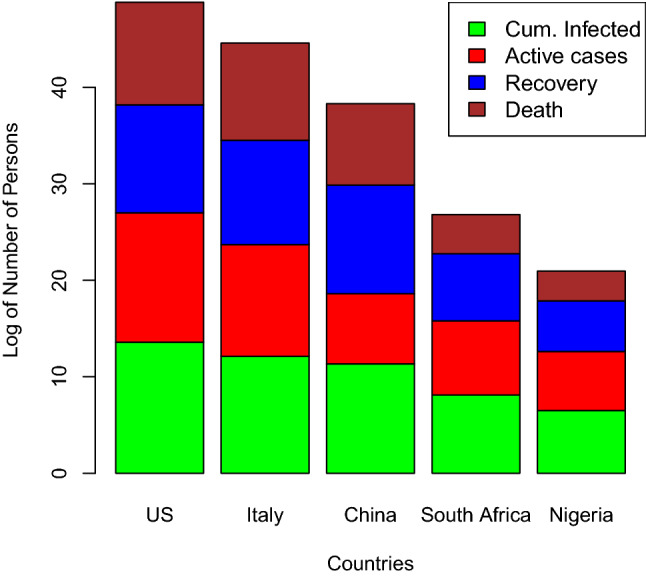
Component bar plot of cum infected, active, recovery, and death of selected countries

Figure 9 shows a component bar plot of confirmed COVID-19 cases, active cases, recovery, and deaths of 5 selected countries out of the 15 under study. China is the only country where cases of recovery is greater than active cases during the period under study. The size of each component of a bar is directly proportional to the quantity measured.

**Fig. 10:**
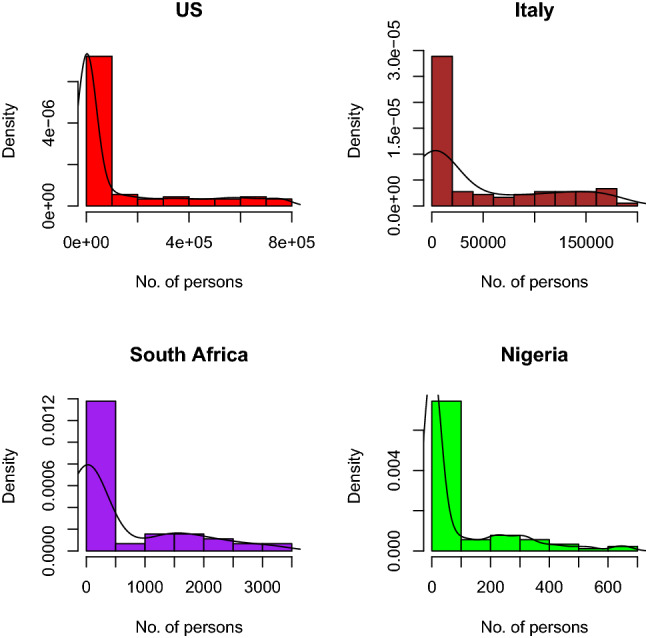
Histogram of confirmed cases of selected countries

Figure 10 shows the histogram of confirmed cases of 4 selected countries. All the histograms show positive skewness. This implies that at the initial start of COVID-19 all of these countries have few cases of COVID-19 as compared to subsequent days. The level of skewness varies from country to country.

**Fig. 11:**
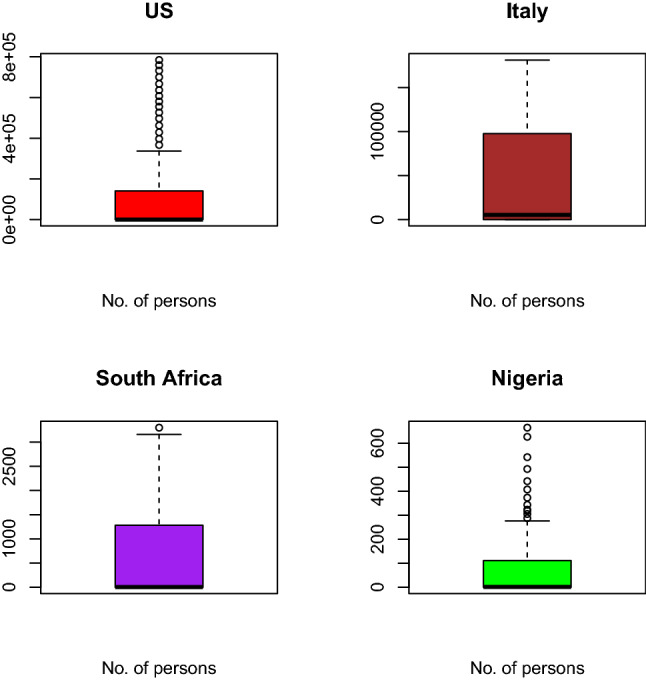
Box plot of confirmed cases of selected countries

Figure 11 shows a box plot showing COVID-19 confirmed cases to buttress Fig. 10. USA and Nigeria have outliers, South Africa as just one outlier while Italy has no outlier. Outliers are extreme values that occur unusually. It makes the mean value to be overestimated or underestimated. The box plot shows the maximum, 1st quartile, median 3rd quartile, and minimum in that order from top to bottom.

**Fig. 12:**
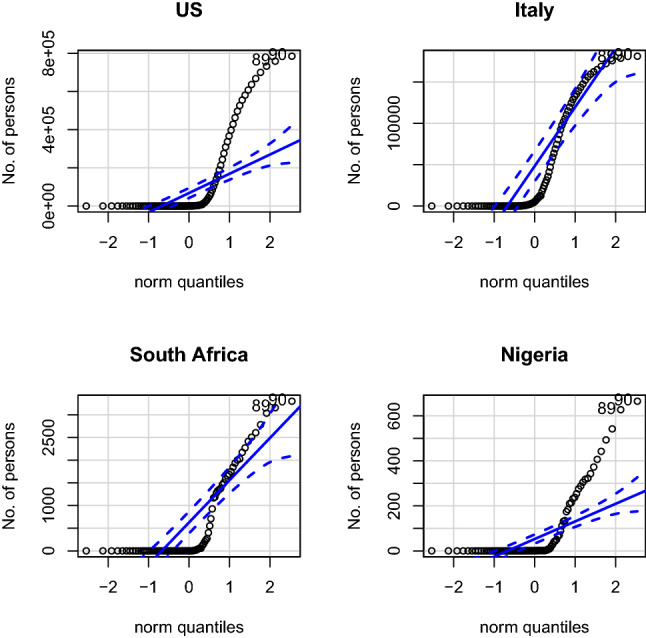
QQplot of confirmed cases of selected countries

Figure 12 shows a QQplot showing that the COVID-19 cumulative infected data are not normally distributed for all the selected countries under study. Any test that depends on normality assumptions cannot be carried out on the data in their current for except they are normalized. However, the cumulative infected curve shows that the curve is becoming flattened for some countries like Italy and then USA. For South Africa and Nigeria, the curves are still steeping up.

**Fig. 13:**
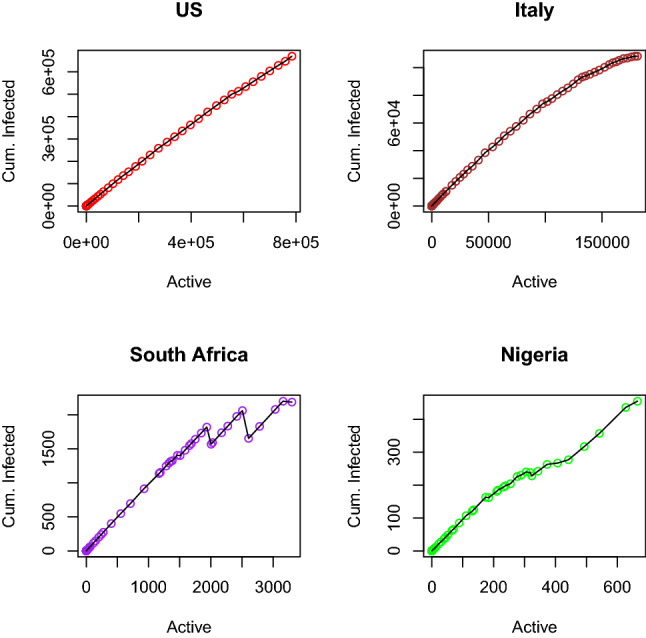
Scatter plot of confirmed cases of selected countries

**Fig. 14:**
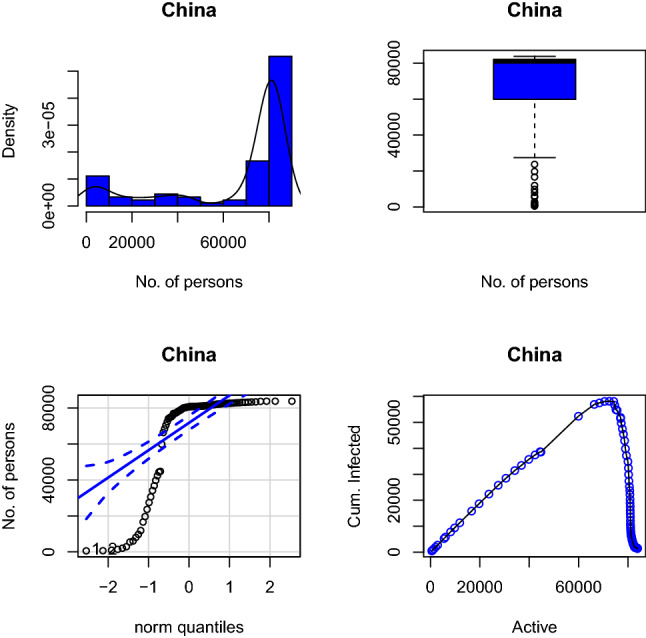
China confirmed infected cases of COVID-19

Figure 13 shows a scatter plot showing the relationship between confirmed infected cases and active cases. For USA, the linear relationship is almost perfect; for Italy the relationship is encouraging. For South Africa, there are three change points, which shows three different situations in the country active cases tend to reduce but increase with new infected cases. In Nigeria, it is not that regular. The ideal situation is that as confirmed cases increase, closed cases should also increase and reduce active cases. This situation is what we have in Fig. 14 for China case.

Four plots depicted in Figs. 10, 11, 12 and 13 are all embedded in Fig. 14 for China only. The histogram here is negatively skewed as against that of other countries in this study. This depicts the infected number cases in China was very high at the beginning of the outbreak, but the rate of increase reduces with time. This is also supported by the box plot with outliers at the bottom as against USA, Nigeria, and South Africa having outliers at the top. China cumulative COVID-19 data are not also normally distributed, it has to be normalized before any analysis that depends on normality assumption can be carried out on it, but the cumulative curve shows that it is flattened at the top. The scatter plot showing the relationship between cumulative infected and active cases depicts that at the initial stage of the China outbreaks, just like any other country, both were increasing, meaning that as the infected cases increase, the active cases also increase. This continue until a peak is reached, so that little increase in cumulative infected cases resulted to a great decrease in active cases and bringing the active cases close to zero. If this curve touches zero, it implies that if individual is infected with the virus, then there is certainty that the individual will recover in no time.

**Fig. 15:**
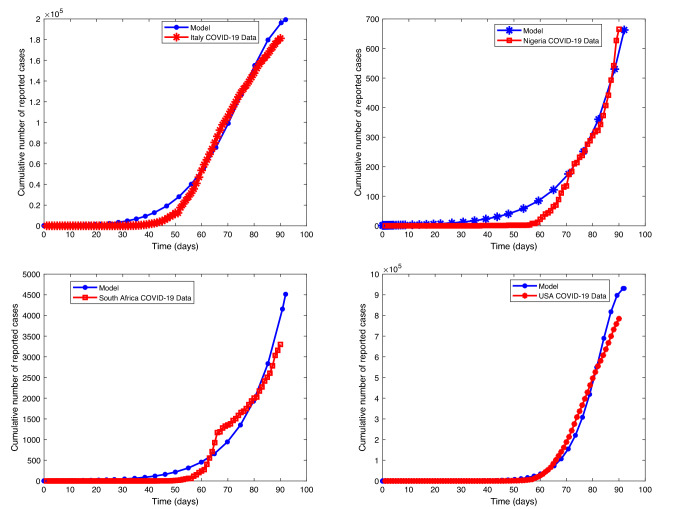
Fitting the cumulative number of reported cases of Selected Countries

Figure 15 shows that our model fitted well with the selected countries data (daily cumulative number of reported cases).

**Fig. 16:**
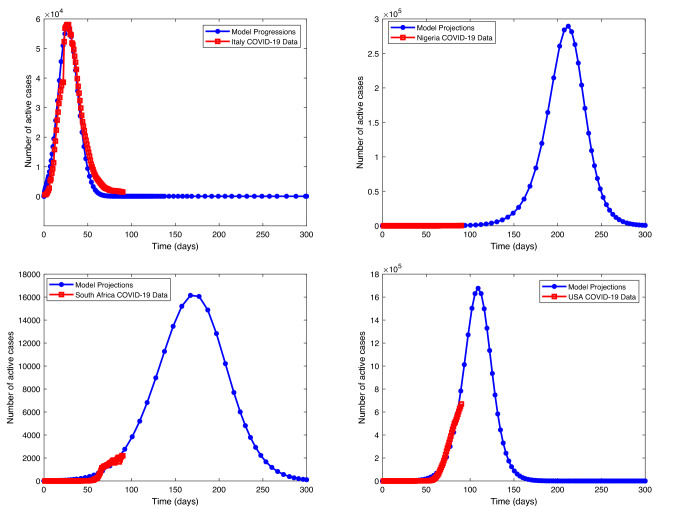
Projection of number of active cases of Selected Countries

Figure 16 (upper left) shows that compliance to physical distancing in Italy will significantly decrease the active number cases in the coming days and eventually eliminate COVID-19 from Italy completely. Figure 16 (upper right) shows that compliance to physical distancing in Nigeria would reduce the active number cases in the coming days. The COVID-19 active number cases would rise to the highest point. The current situation shows that active cases might rise, but with compliance to physical distancing, COVID-19 would decrease to zero in as time increases. The active cases are the number of individuals that are currently infected with COVID-19 and are still alive. It is the cumulative infected cases—closed cases, where closed cases are the number of deaths and recovery. So, as the recovery number is raised, the active number cases reduce. Italy active cases are far below their maximum actives cases, but that of USA, South Africa, and Nigeria have not reached their pick as at the time of this report. Figure 16 (lower left) shows that compliance to physical distancing in South Africa will reduce the active number cases in the coming days. The COVID-19 active number cases would rise to a highest point, but may not go beyond 17,000 active cases in the worst scenario after which it decreases to zero as time increases. Figure 16 (lower right) shows that compliance to physical distancing in USA will reduce the number of active cases in the coming days. The active cases of COVID-19 would increase to a maximum point, almost twice the current number for the period under review, after which it decreases to zero with time.

## Optimal control problem setup

As presented in the earlier sections, the quarantine and compliance to physical distancing play high roles in decreasing the affected COVID-19 individual numbers. Meanwhile, it was noticed that nations with lowest compliance will result to increase recruitment rate. Hence, it is recommended that in a case of rising inflow individuals, vaccination must be encouraged. Therefore, an optimal control is formulated to manage optimal trajectories by embedding vaccination intervention (i.e., pharmaceutical) in the model 2.6. As such, objective functions *J* are developed containing $$u_{1}(t)> 0, u_{2}(t) > 0 $$. Our $$ u_{1}(t)$$ represents the public health advise on the usages of non-pharmaceutical intervention without or with vaccine (such as wearing of face mask, social/physical distancing, washing hands often, staying home when sick, canceling or postponing mass gatherings, making sick leave policies more flexible, remote meeting or offering tele-work options, self-isolation reduced COVID-19 spread). While $$ u_{2}(t)$$ is considered as the cost functional control variable, a quadratic function for control and state is chosen, which takes into consideration the infectious individual and vaccination consumption numbers. Thus, the weight constant of the Quarantine $$Q_{h}(t)$$, and Infected $$I_{h}(t)$$, is represented by $$A_{1}$$ and $$A_{2}$$. Likewise, the weight constants $$\omega _{1}$$ and $$\omega _{2}$$ are related to interventions cost different over a finite time period T, the transmission decreasing rate due to non-pharmaceutical and pharmaceutical. Now, using the control time-dependent, a modified equation of the formulation 2.6 is gotten as

### COVID-19 model with control

5.1 $$\begin{aligned} \frac{\mathrm{{d}}S_{h}}{\mathrm{{d}}t}= & {} \Pi +\alpha M +\rho V_{h}+\delta _{0} \left( 1-p_{1}\right) Q_{h}+\delta _{1} R_{h}\nonumber \\{} & {} -\left( \frac{\left( 1-u_{2}\right) p_{1} \beta I_{h}}{S_{h}+V_{h}+Q_{h}+I_{h}+R_{h}}+u_{1}+\tau +\mu \right) S_{h}\nonumber \\ \frac{\mathrm{{d}}V_{h}}{\mathrm{{d}}t}= & {} u_{1} S_{h}+u_{1} R_{h}\nonumber \\{} & {} -\left( \frac{\left( 1-\varepsilon \right) \left( 1-u_{2}\right) p_{1} \beta I_{h}}{S_{h}+V_{h}+Q_{h}+I_{h}+R_{h}}+\rho +\mu \right) V_{h} \nonumber \\ \frac{\mathrm{{d}}Q_{h}}{\mathrm{{d}}t}= & {} \frac{\left( S_{h}+\left( 1-\varepsilon \right) V_{h}\right) \left( 1-u_{2}\right) p_{1} \beta I_{h}}{S_{h}+V_{h}+Q_{h}+I_{h}+R_{h}}\nonumber \\{} & {} -\left( \theta +\delta _{0} \left( 1-p_{1}\right) +\mu \right) Q_{h} \nonumber \\ \frac{\mathrm{{d}}I_{h}}{\mathrm{{d}}t}= & {} \theta Q_{h}-\left( \gamma +\sigma +\mu \right) I_{h} \nonumber \\ \frac{\mathrm{{d}}_{h}}{\mathrm{{d}}t}= & {} \gamma I_{h}+\tau S_{h}-\left( u_{1}+\delta _{1}+\mu \right) R_{h} \end{aligned}$$

with corresponding objective functional

$$\begin{aligned} J = \min \int \left( w_{1} u_{1}^{2}+w_{2} u_{2}^{2}+A_{1} I_{h}+A_{2} Q_{h}\right) \mathrm{{d}}t \end{aligned}$$

### Determination of the necessary conditions for optimality

The optimal control necessary conditions satisfy the adjoint equations and optimality solutions, which come from Pontryagin’s Maximum Principle [19, 42, 53]. This principle converts systems 5.1 to a minimizing point-wise of Hamiltonian function $\mathcal {H}$ form that is obtained when each state variables correspond to adjoint variables and combining with the objective functional results.

5.2 $$\begin{aligned} J = \min \int \left( w_{1} u_{1}^{2}+w_{2} u_{2}^{2}+A_{1} I_{h}+A_{2} Q_{h}\right) \mathrm{{d}} t \end{aligned}$$

where $u_{1}$ $\epsilon$ *U* is Lebesque measurable, which can be defined as

$$\theta =\left\{ 0\le u_{i}(t) \le 1, \quad \mathrm{{for}} \quad i=1,2 \quad \mathrm{{and }} \quad t \in [0, T] \right\} ,$$

The resulted equation is obtained

5.3 $$\begin{aligned} \mathcal {H}(t, y, u, \theta )= & {} w_{1} u_{1}^{2}+w_{2} u_{2}^{2}+\theta _{1} B1 +\theta _{2} B2 \nonumber \\{} & {} +\theta _{3} B3 +\theta _{4} B4 +\theta _{5} B5 +A_{1} i_{h}+A_{2} Q_{h}\nonumber \\ \end{aligned}$$

where

$\theta _{i}$ for $i=1,...,5$ are the associated adjoint functions with the state equations of (5.1), on the RHS of the *ith* state variable of the system of derivatives (5.1). The extended state of the Hamiltonian function of (5.3) is expressed as

5.4 $$\begin{aligned}{} & {} \mathcal {H}(t, y, u, \lambda )=w_{1} u_{1}^{2}+w_{2} u_{2}^{2}+ A_{1}i_{h}+A_{2}Q_{h}+ \theta _{1} B1 \nonumber \\{} & {} \qquad +\theta _{2} B2 +\theta _{3} B3 +\theta _{4} B4 +\theta _{5} B5 \nonumber \\{} & {} \qquad + \left\{ \Pi +\alpha M +\rho V_{h}+\delta _{0} \left( 1-p_{1}\right) Q_{h}+\delta _{1} R_{h}\right. \nonumber \\{} & {} \qquad \left. -\left( \frac{\left( 1-u_{2}\right) p_{1} \beta I_{h}}{S_{h}+V_{h}+Q_{h}+I_{h}+R_{h}}+u_{1}+\tau +\mu \right) S_{h}\right\} \theta _{1} \nonumber \\{} & {} \qquad + \left\{ u_{1} S_{h}+u_{1} R_{h}-\left( \frac{\left( 1-\varepsilon \right) \left( 1-u_{2}\right) p_{1} \beta I_{h}}{S_{h}+V_{h}+Q_{h}+I_{h}+R_{h}}\right. \right. \nonumber \\{} & {} \qquad \left. \left. +\rho +\mu \right) V_{h} \right\} \theta _{2} \nonumber \\{} & {} \qquad + \left\{ \frac{\left( S_{h}+\left( 1-\varepsilon \right) V_{h}\right) \left( 1-u_{2}\right) p_{1} \beta I_{h}}{S_{h}+V_{h}+Q_{h}+I_{h}+R_{h}}\right. \nonumber \\{} & {} \qquad \left. -\left( \theta +\delta _{0} \left( 1-p_{1}\right) +\mu \right) Q_{h} \right\} \theta _{3} \nonumber \\{} & {} \qquad + \left\{ \theta Q_{h}-\left( \gamma +\sigma +\mu \right) I_{h}\right\} \theta _{4} \end{aligned}$$

5.5 $$\begin{aligned}{} & {} \qquad + \left\{ \gamma I_{h}+\tau S_{h}-\left( u_{1}+\delta _{1}+\mu \right) R_{h} \right\} \theta _{5} \end{aligned}$$

### Hamiltonian partial derivative for obtaining the adjoint variables in respect to each state variables

In respect to the respective control variables $(u_{1},u_{2})$, the optimality equations are gotten through a partial differentiation of the Hamiltonian function $\mathcal {H}$. The adjoint equation $\theta ^{\prime }$ in time derivative is gotten by considering the non-positive partial differentiation of $\mathcal {H}$ in respect to state variables *y*(*t*) model so that $\theta ^{\prime }=-\mathcal {H}$.

5.6 $$\begin{aligned}{} & {} \begin{aligned} \frac{\textrm{d}{\theta _{1}}}{\textrm{d}{t}} =&-\left( \frac{\left( 1-u_{2}\right) p_{1} \beta i_{h} S_{h}}{\left( S_{h}+V_{h}+Q_{h}+i_{h}+R_{h}\right) ^{2}}\right. \\&\left. -\frac{\left( 1-u_{2}\right) p_{1} \beta i_{h}}{S_{h}+V_{h}+Q_{h}+i_{h}+R_{h}}-u_{1}-\tau -\mu \right) \theta _{1}\\&-\left( u_{1}+\frac{\left( 1-\varepsilon \right) \left( 1-u_{2}\right) p_{1} \beta i_{h} V_{h}}{\left( S_{h}+V_{h}+Q_{h}+i_{h}+R_{h}\right) ^{2}}\right) \theta _{2}\\&-\left( \frac{\left( 1-u_{2}\right) p_{1} \beta i_{h}}{S_{h}+V_{h}+Q_{h}+i_{h}+R_{h}}\right. \\&\left. -\frac{\left( S_{h}+\left( 1-\varepsilon \right) V_{h}\right) \left( 1-u_{2}\right) p_{1} \beta i_{h}}{\left( S_{h}+V_{h}+Q_{h}+i_{h}+R_{h}\right) ^{2}}\right) \theta _{3}-\tau \theta _{5} \end{aligned} \end{aligned}$$

5.7 $$\begin{aligned}{} & {} \begin{aligned} \frac{\textrm{d}{\theta _{2}}}{\textrm{d}{t}} =&-\left( \rho +\frac{\left( 1-u_{2}\right) p_{1} \beta i_{h} S_{h}}{\left( S_{h}+V_{h}+Q_{h}+i_{h}+R_{h}\right) ^{2}}\right) \theta _{1}\\&-\left( \frac{\left( 1-\varepsilon \right) \left( 1-u_{2}\right) p_{1} \beta i_{h} V_{h}}{\left( S_{h}+V_{h}+Q_{h}+i_{h}+R_{h}\right) ^{2}}\right. \\&\left. -\frac{\left( 1-\varepsilon \right) \left( 1-u_{2}\right) p_{1} \beta i_{h}}{S_{h}+V_{h}+Q_{h}+i_{h}+R_{h}}-\rho -\mu \right) \theta _{2}\\&-\left( \frac{\left( 1-\varepsilon \right) \left( 1-u_{2}\right) p_{1} \beta i_{h}}{S_{h}+V_{h}+Q_{h}+i_{h}+R_{h}}\right. \\&\left. -\frac{\left( S_{h}+\left( 1-\varepsilon \right) V_{h}\right) \left( 1-u_{2}\right) p_{1} \beta i_{h}}{\left( S_{h}+V_{h}+Q_{h}+i_{h}+R_{h}\right) ^{2}}\right) \theta _{3} \end{aligned} \end{aligned}$$

5.8 $$\begin{aligned}{} & {} \begin{aligned} \frac{\textrm{d}{\theta _{3}}}{\textrm{d}{t}} =&-\left( \delta _{0} \left( 1-p_{1}\right) +\frac{\left( 1-u_{2}\right) p_{1} \beta i_{h} S_{h}}{\left( S_{h}+V_{h}+Q_{h}+i_{h}+R_{h}\right) ^{2}}\right) \theta _{1}\\&-\frac{\left( 1-\varepsilon \right) \left( 1-u_{2}\right) p_{1} \beta i_{h} V_{h} \theta _{2}}{\left( S_{h}+V_{h}+Q_{h}+i_{h}+R_{h}\right) ^{2}}\\&-\left( -\frac{\left( S_{h}+\left( 1-\varepsilon \right) V_{h}\right) \left( 1-u_{2}\right) p_{1} \beta i_{h}}{\left( S_{h}+V_{h}+Q_{h}+i_{h}+R_{h}\right) ^{2}}\right. \\&\left. -\theta -\delta _{0} \left( 1-p_{1}\right) -\mu \right) \theta _{3}-\theta \theta _{4}-A_{2} \end{aligned} \end{aligned}$$

5.9 $$\begin{aligned}{} & {} \begin{aligned} \frac{\textrm{d}{\theta _{4}}}{\textrm{d}{t}} =&\left( \frac{\left( 1-u_{2}\right) p_{1} \beta }{S_{h}+V_{h}+Q_{h}+i_{h}+R_{h}}\right. \\&\left. -\frac{\left( 1-u_{2}\right) p_{1} \beta i_{h}}{\left( S_{h}+V_{h}+Q_{h}+i_{h}+R_{h}\right) ^{2}}\right) S_{h} \theta _{1}\\&+\left( \frac{\left( 1-\varepsilon \right) \left( 1-u_{2}\right) p_{1} \beta }{S_{h}+V_{h}+Q_{h}+i_{h}+R_{h}}\right. \\&\left. -\frac{\left( 1-\varepsilon \right) \left( 1-u_{2}\right) p_{1} \beta i_{h}}{\left( S_{h}+V_{h}+Q_{h}+i_{h}+R_{h}\right) ^{2}}\right) V_{h} \theta _{2}\\&-\left( \frac{\left( S_{h}+\left( 1-\varepsilon \right) V_{h}\right) \left( 1-u_{2}\right) p_{1}\beta }{S_{h}+V_{h}+Q_{h}+i_{h}+R_{h}}\right. \\&\left. -\frac{\left( S_{h}+\left( 1-\varepsilon \right) V_{h}\right) \left( 1-u_{2}\right) p_{1} \beta i_{h}}{\left( S_{h}+V_{h}+Q_{h}+i_{h}+R_{h}\right) ^{2}}\right) \theta _{3}\\&-\left( -\gamma -\sigma -\mu \right) \theta _{4}-\gamma \theta _{5}-A_{1} \end{aligned} \end{aligned}$$

5.10 $$\begin{aligned}{} & {} \begin{aligned} \frac{\textrm{d}{\theta _{5}}}{\textrm{d}{t}} =&-\left( \delta _{1}+\frac{\left( 1-u_{2}\right) p_{1} \beta i_{h} S_{h}}{\left( S_{h}+V_{h}+Q_{h}+i_{h}+R_{h}\right) ^{2}}\right) \theta _{1}\\&-\left( u_{1}+\frac{\left( 1-\varepsilon \right) \left( 1-u_{2}\right) p_{1} \beta i_{h} V_{h}}{\left( S_{h}+V_{h}+Q_{h}+i_{h}+R_{h}\right) ^{2}}\right) \theta _{2}\\&+\frac{\left( S_{h}+\left( 1-\varepsilon \right) V_{h}\right) \left( 1-u_{2}\right) p_{1} \beta i_{h} \theta _{3}}{\left( S_{h}+V_{h}+Q_{h}+i_{h}+R_{h}\right) ^{2}}\\&-\left( -u_{1}-\delta _{1}-\mu \right) \theta _{5} \end{aligned} \end{aligned}$$

#### Theorem 3

There exist a set $(u_{1}^{*},u_{2}^{*})$ of an optimal control and their consequential state solutions $S_{h}^{*},V_{h}^{*}, Q_{h}^{*}, I_{h}^{*}$ and $R_{h}^{*}$ that minimize $\mathcal {J}(u_{1},u_{2})$, and therefore, there exist adjoint functions $\theta _{1}, \lambda _{2},...,\lambda _{5}$ such that

5.11 $$\begin{aligned}{} & {} \begin{aligned} \frac{\textrm{d}{\theta _{1}}}{\textrm{d}{t}} =&-\left( \frac{\left( 1-u_{2}\right) p_{1} \beta i_{h} S_{h}}{\left( S_{h}+V_{h}+Q_{h}+i_{h}+R_{h}\right) ^{2}}\right. \\&\left. -\frac{\left( 1-u_{2}\right) p_{1} \beta i_{h}}{S_{h}+V_{h}+Q_{h}+i_{h}+R_{h}}-u_{1}-\tau -\mu \right) \theta _{1}\\&-\left( u_{1}+\frac{\left( 1-\varepsilon \right) \left( 1-u_{2}\right) p_{1} \beta i_{h}V_{h}}{\left( S_{h}+V_{h}+Q_{h}+i_{h}+R_{h}\right) ^{2}}\right) \theta _{2}\\&-\left( \frac{\left( 1-u_{2}\right) p_{1} \beta i_{h}}{S_{h}+V_{h}+Q_{h}+i_{h}+R_{h}}\right. \\&\left. -\frac{\left( S_{h}+\left( 1-\varepsilon \right) V_{h}\right) \left( 1-u_{2}\right) p_{1} \beta i_{h}}{\left( S_{h}+V_{h}+Q_{h}+i_{h}+R_{h}\right) ^{2}}\right) \theta _{3}-\tau \theta _{5} \end{aligned} \nonumber \\{} & {} \begin{aligned} \frac{\textrm{d}{\theta _{2}}}{\textrm{d}{t}}\theta _{2} =&-\left( \rho +\frac{\left( 1-u_{2}\right) p_{1} \beta i_{h} S_{h}}{\left( S_{h}+V_{h}+Q_{h}+i_{h}+R_{h}\right) ^{2}}\right) \theta _{1}\\&-\left( \frac{\left( 1-\varepsilon \right) \left( 1-u_{2}\right) p_{1} \beta i_{h}V_{h}}{\left( S_{h}+V_{h}+Q_{h}+i_{h}+R_{h}\right) ^{2}}\right. \\&\left. -\frac{\left( 1-\varepsilon \right) \left( 1-u_{2}\right) p_{1} \beta i_{h}}{S_{h}+V_{h}+Q_{h}+i_{h}+R_{h}}-\rho -\mu \right) \theta _{2}\\&-\left( \frac{\left( 1-\varepsilon \right) \left( 1-u_{2}\right) p_{1} \beta i_{h}}{S_{h}+V_{h}+Q_{h}+i_{h}+R_{h}}\right. \\&\left. -\frac{\left( S_{h}+\left( 1-\varepsilon \right) V_{h}\right) \left( 1-u_{2}\right) p_{1} \beta i_{h}}{\left( S_{h}+V_{h}+Q_{h}+i_{h}+R_{h}\right) ^{2}}\right) \theta _{3} \end{aligned} \nonumber \\{} & {} \begin{aligned} \frac{\textrm{d}{\theta _{3}}}{\textrm{d}{t}} =&-\left( \delta _{0} \left( 1-p_{1}\right) +\frac{\left( 1-u_{2}\right) p_{1} \beta i_{h} S_{h}}{\left( S_{h}+V_{h}+Q_{h}+i_{h}+R_{h}\right) ^{2}}\right) \theta _{1}\\&-\frac{\left( 1-\varepsilon \right) \left( 1-u_{2}\right) p_{1} \beta i_{h} V_{h} \theta _{2}}{\left( S_{h}+V_{h}+Q_{h}+i_{h}+R_{h}\right) ^{2}}\\&-\left( -\frac{\left( S_{h}+\left( 1-\varepsilon \right) V_{h}\right) \left( 1-u_{2}\right) p_{1} \beta i_{h}}{\left( S_{h}+V_{h}+Q_{h}+i_{h}+R_{h}\right) ^{2}}\right. \\&\left. -\theta -\delta _{0} \left( 1-p_{1}\right) -\mu \right) \theta _{3}-\theta \theta _{4}-A_{2} \end{aligned} \nonumber \\{} & {} \begin{aligned} \frac{\textrm{d}{\theta _{4}}}{\textrm{d}{t}} =&\left( \frac{\left( 1-u_{2}\right) p_{1} \beta }{S_{h}+V_{h}+Q_{h}+i_{h}+R_{h}}\right. \\&\left. -\frac{\left( 1-u_{2}\right) p_{1} \beta i_{h}}{\left( S_{h}+V_{h}+Q_{h}+i_{h}+R_{h}\right) ^{2}}\right) S_{h} \theta _{1}\\&+\left( \frac{\left( 1-\varepsilon \right) \left( 1-u_{2}\right) p_{1} \beta }{S_{h}+V_{h}+Q_{h}+i_{h}+R_{h}}\right. \\&\left. -\frac{\left( 1-\varepsilon \right) \left( 1-u_{2}\right) p_{1} \beta i_{h}}{\left( S_{h}+V_{h}+Q_{h}+i_{h}+R_{h}\right) ^{2}}\right) V_{h}\theta _{2}\\&-\left( \frac{\left( S_{h}+\left( 1-\varepsilon \right) V_{h}\right) \left( 1-u_{2}\right) p_{1} \beta }{S_{h}+V_{h}+Q_{h}+i_{h}+R_{h}}\right. \\&\left. -\frac{\left( S_{h}+\left( 1-\varepsilon \right) V_{h}\right) \left( 1-u_{2}\right) p_{1} \beta i_{h}}{\left( S_{h}+V_{h}+Q_{h}+i_{h}+R_{h}\right) ^{2}}\right) \theta _{3}\\&-\left( -\gamma -\sigma -\mu \right) \theta _{4}-\gamma \theta _{5}-A_{1} \end{aligned} \nonumber \\{} & {} \begin{aligned} \frac{\textrm{d}{\theta _{5}}}{\textrm{d}{t}} =&-\left( \delta _{1}+\frac{\left( 1-u_{2}\right) p_{1} \beta i_{h} S_{h}}{\left( S_{h}+V_{h}+Q_{h}+i_{h}+R_{h}\right) ^{2}}\right) \theta _{1}\\&-\left( u_{1}+\frac{\left( 1-\varepsilon \right) \left( 1-u_{2}\right) p_{1} \beta i_{h} V_{h}}{\left( S_{h}+V_{h}+Q_{h}+i_{h}+R_{h}\right) ^{2}}\right) \theta _{2}\\&+\frac{\left( S_{h}+\left( 1-\varepsilon \right) V_{h}\right) \left( 1-u_{2}\right) p_{1} \beta i_{h}\theta _{3}}{\left( S_{h}+V_{h}+Q_{h}+i_{h}+R_{h}\right) ^{2}}\\&-\left( -u_{1}-\delta _{1}-\mu \right) \theta _{5} \end{aligned} \end{aligned}$$

with $\left\{ \lambda _{i}(T) \quad for \quad i=1,2,...,5\right\} =0$ transversality conditions, and the control variable $(u_{1}^{*}, u_{2}^{*})$, satisfies the following optimality conditions:

5.12 $$\begin{aligned} u_{1}^{*}= & {} \min \left\{ \max \left\{ 0, \quad -\frac{\theta _{2} R_{h}-R_{h} \theta _{5}-S_{h} \theta _{1}+\theta _{2} S_{h}}{2 w_{1}} \right\} , \quad 1 \right\} \nonumber \\ u_{2}^{*}= & {} \min \left\{ \max \left\{ 0, \quad \right. \right. \nonumber \\{} & {} \left. \left. -\frac{p_{1} \beta i_{h} \left( -\varepsilon V_{h} \theta _{2}+\varepsilon V_{h} \theta _{3}+S_{h} \theta _{1}-S_{h} \theta _{3}+V_{h} \theta _{2}-V_{h} \theta _{3}\right) }{2 \left( S_{h}+V_{h}+Q_{h}+i_{h}+R_{h}\right) w_{2}} \right\} , \quad 1 \right\} \nonumber \\ \end{aligned}$$

#### Proof

Consider the optimal control $u^{*}=(u_{1}^{*}, u_{2}^{*})$ and $S_{h}^{*},V_{h}^{*}, Q_{h}^{*}, I_{h}^{*},$ and $R_{h}^{*}$ is the respective state variable model. Using the Pontryagin’s Maximum Principle, adjoint variables are satisfied as:

5.13 $$\begin{aligned} \frac{\mathrm{{d}}\theta _{1}}{\mathrm{{d}}t}= & {} -\frac{\partial H}{\partial S_{h}}, \qquad \theta _{1}(t_{f})=0 \nonumber \\ \frac{\mathrm{{d}}\theta _{2}}{\mathrm{{d}}t}= & {} -\frac{\partial H}{\partial V_{h}}, \qquad \theta _{2}(t_{f})=0 \nonumber \\ \frac{\mathrm{{d}}\theta _{3}}{\mathrm{{d}}t}= & {} -\frac{\partial H}{\partial Q_{h}}, \qquad \theta _{3}(t_{f})=0 \nonumber \\ \frac{\mathrm{{d}}\theta _{4}}{\mathrm{{d}}t}= & {} -\frac{\partial H}{\partial I_{h}}, \qquad \theta _{4}(t_{f})=0 \end{aligned}$$

5.14 $$\begin{aligned} \frac{\mathrm{{d}}\theta _{5}}{\mathrm{{d}}t}= & {} -\frac{\partial H}{\partial R_{h}}, \qquad \theta _{5}(t_{f})=0 \end{aligned}$$

with $\theta _{i}(t_{f})=0$, for $i=1,2,...5$ transversality conditions. Therefore, at the optimal controls $u_{1}$ and $u_{2}$, the adjoint system is determined and the respective state variables model $S_{h}, V_{h}, Q_{h}, I_{h}, R_{h}$ is as given by

5.15 $$\begin{aligned}{} & {} \begin{aligned} \frac{\textrm{d}{\theta _{1}}}{\textrm{d}{t}} =&-\left( \frac{\left( 1-u_{2}\right) p_{1} \beta i_{h} S_{h}}{\left( S_{h}+V_{h}+Q_{h}+i_{h}+R_{h}\right) ^{2}}\right. \\&\left. -\frac{\left( 1-u_{2}\right) p_{1} \beta i_{h}}{S_{h}+V_{h}+Q_{h}+i_{h}+R_{h}}-u_{1}-\tau -\mu \right) \theta _{1}\\&-\left( u_{1}+\frac{\left( 1-\varepsilon \right) \left( 1-u_{2}\right) p_{1} \beta i_{h} V_{h}}{\left( S_{h}+V_{h}+Q_{h}+i_{h}+R_{h}\right) ^{2}}\right) \theta _{2}\\&-\left( \frac{\left( 1-u_{2}\right) p_{1} \beta i_{h}}{S_{h}+V_{h}+Q_{h}+i_{h}+R_{h}}\right. \\&\left. -\frac{\left( S_{h}+\left( 1-\varepsilon \right) V_{h}\right) \left( 1-u_{2}\right) p_{1} \beta i_{h}}{\left( S_{h}+V_{h}+Q_{h}+i_{h}+R_{h}\right) ^{2}}\right) \theta _{3}-\tau \theta _{5} \end{aligned} \nonumber \\{} & {} \begin{aligned} \frac{\textrm{d}{\theta _{2}}}{\textrm{d}{t}}\theta _{2} =&-\left( \rho +\frac{\left( 1-u_{2}\right) p_{1} \beta i_{h} S_{h}}{\left( S_{h}+V_{h}+Q_{h}+i_{h}+R_{h}\right) ^{2}}\right) \theta _{1}\\&-\left( \frac{\left( 1-\varepsilon \right) \left( 1-u_{2}\right) p_{1} \beta i_{h}V_{h}}{\left( S_{h}+V_{h}+Q_{h}+i_{h}+R_{h}\right) ^{2}}\right. \\&\left. -\frac{\left( 1-\varepsilon \right) \left( 1-u_{2}\right) p_{1} \beta i_{h}}{S_{h}+V_{h}+Q_{h}+i_{h}+R_{h}}-\rho -\mu \right) \theta _{2}\\&-\left( \frac{\left( 1-\varepsilon \right) \left( 1-u_{2}\right) p_{1} \beta i_{h}}{S_{h}+V_{h}+Q_{h}+i_{h}+R_{h}}\right. \\&\left. -\frac{\left( S_{h}+\left( 1-\varepsilon \right) V_{h}\right) \left( 1-u_{2}\right) p_{1} \beta i_{h}}{\left( S_{h}+V_{h}+Q_{h}+i_{h}+R_{h}\right) ^{2}}\right) \theta _{3} \end{aligned} \nonumber \\{} & {} \begin{aligned} \frac{\textrm{d}{\theta _{3}}}{\textrm{d}{t}} =&-\left( \delta _{0} \left( 1-p_{1}\right) +\frac{\left( 1-u_{2}\right) p_{1} \beta i_{h} S_{h}}{\left( S_{h}+V_{h}+Q_{h}+i_{h}+R_{h}\right) ^{2}}\right) \theta _{1}\\&-\frac{\left( 1-\varepsilon \right) \left( 1-u_{2}\right) p_{1} \beta i_{h}V_{h}\theta _{2}}{\left( S_{h}+V_{h}+Q_{h}+i_{h}+R_{h}\right) ^{2}}\\&-\left( -\frac{\left( S_{h}+\left( 1-\varepsilon \right) V_{h}\right) \left( 1-u_{2}\right) p_{1} \beta i_{h}}{\left( S_{h}+V_{h}+Q_{h}+i_{h}+R_{h}\right) ^{2}}\right. \\&\left. -\theta -\delta _{0} \left( 1-p_{1}\right) -\mu \right) \theta _{3}-\theta \theta _{4}-A_{2} \end{aligned} \nonumber \\{} & {} \begin{aligned} \frac{\textrm{d}{\theta _{4}}}{\textrm{d}{t}} =&\left( \frac{\left( 1-u_{2}\right) p_{1} \beta }{S_{h}+V_{h}+Q_{h}+i_{h}+R_{h}}\right. \\&\left. -\frac{\left( 1-u_{2}\right) p_{1} \beta i_{h}}{\left( S_{h}+V_{h}+Q_{h}+i_{h}+R_{h}\right) ^{2}}\right) S_{h} \theta _{1}\\&+\left( \frac{\left( 1-\varepsilon \right) \left( 1-u_{2}\right) p_{1} \beta }{S_{h}+V_{h}+Q_{h}+i_{h}+R_{h}}\right. \\&\left. -\frac{\left( 1-\varepsilon \right) \left( 1-u_{2}\right) p_{1} \beta i_{h}}{\left( S_{h}+V_{h}+Q_{h}+i_{h}+R_{h}\right) ^{2}}\right) V_{h}\theta _{2}\\&-\left( \frac{\left( S_{h}+\left( 1-\varepsilon \right) V_{h}\right) \left( 1-u_{2}\right) p_{1} \beta }{S_{h}+V_{h}+Q_{h}+i_{h}+R_{h}}\right. \\&\left. -\frac{\left( S_{h}+\left( 1-\varepsilon \right) V_{h}\right) \left( 1-u_{2}\right) p_{1} \beta i_{h}}{\left( S_{h}+V_{h}+Q_{h}+i_{h}+R_{h}\right) ^{2}}\right) \theta _{3}\\&-\left( -\gamma -\sigma -\mu \right) \theta _{4}-\gamma \theta _{5}-A_{1} \end{aligned} \nonumber \\{} & {} \begin{aligned} \frac{\textrm{d}{\theta _{5}}}{\textrm{d}{t}} =&-\left( \delta _{1}+\frac{\left( 1-u_{2}\right) p_{1} \beta i_{h} S_{h}}{\left( S_{h}+V_{h}+Q_{h}+i_{h}+R_{h}\right) ^{2}}\right) \theta _{1}\\&-\left( u_{1}+\frac{\left( 1-\varepsilon \right) \left( 1-u_{2}\right) p_{1} \beta i_{h} V_{h}}{\left( S_{h}+V_{h}+Q_{h}+i_{h}+R_{h}\right) ^{2}}\right) \theta _{2}\\&+\frac{\left( S_{h}+\left( 1-\varepsilon \right) V_{h}\right) \left( 1-u_{2}\right) p_{1} \beta i_{h} \theta _{3}}{\left( S_{h}+V_{h}+Q_{h}+i_{h}+R_{h}\right) ^{2}}\\&-\left( -u_{1}-\delta _{1}-\mu \right) \theta _{5} \end{aligned} \end{aligned}$$

with $\left\{ \theta _{i}(T) \quad for \quad i=1,2,3,..,5\right\} =0$ transversality conditions, and the optimal controls $u_{1}^{*}, u_{2}^{*}$ characterized, the optimality equations follow the conditions:

5.16 $$\begin{aligned} \frac{\partial H}{\partial u_{1}}= \frac{\partial H}{\partial u_{2}}= 0 \end{aligned}$$

subject to (5.16), the optimality condition gives the control Lebesque measurable set

$$\begin{aligned} \theta =\left\{ 0\le u_{i}(t) \le 1, \quad \mathrm{{for}} \quad i=1,2 \quad \mathrm{{and}} \quad t \in [0, T] \right\} , \end{aligned}$$

where the control variables $$u_{1}, u_{2} $$ are measurable functions expressed as

5.17 $$\begin{aligned}{} & {} \frac{\partial H}{\partial u_{1}}= -\frac{\theta _{2} R_{h}-R_{h} \theta _{5}-S_{h} \theta _{1}+\theta _{2} S_{h}}{2 w_{1}} \nonumber \\{} & {} \frac{\partial H}{\partial u_{2}}\nonumber \\{} & {} \quad = -\frac{p_{1} \beta i_{h} \left( -\varepsilon V_{h} \theta _{2}+\varepsilon V_{h} \theta _{3}+S_{h} \theta _{1}-S_{h} \theta _{3}+V_{h} \theta _{2}-V_{h} \theta _{3}\right) }{2 \left( S_{h}+V_{h}+Q_{h}+i_{h}+R_{h}\right) w_{2}} \nonumber \\ \end{aligned}$$

5.18 $$\begin{aligned} u_{1}^{*}= & {} \min \left\{ \max \left\{ 0, \quad -\frac{\theta _{2} R_{h}-R_{h} \theta _{5}-S_{h} \theta _{1}+\theta _{2} S_{h}}{2 w_{1}} \right\} , \quad 1 \right\} \nonumber \\ u_{2}^{*}= & {} \min \left\{ \max \left\{ 0, \quad \right. \right. \nonumber \\{} & {} \left. \left. -\frac{p_{1} \beta i_{h} \left( -\varepsilon V_{h} \theta _{2}+\varepsilon V_{h} \theta _{3}+S_{h} \theta _{1}-S_{h} \theta _{3}+V_{h} \theta _{2}-V_{h} \theta _{3}\right) }{2 \left( S_{h}+V_{h}+Q_{h}+i_{h}+R_{h}\right) w_{2}} \right\} , \quad 1 \right\} \nonumber \\ \end{aligned}$$

**Fig. 17:**
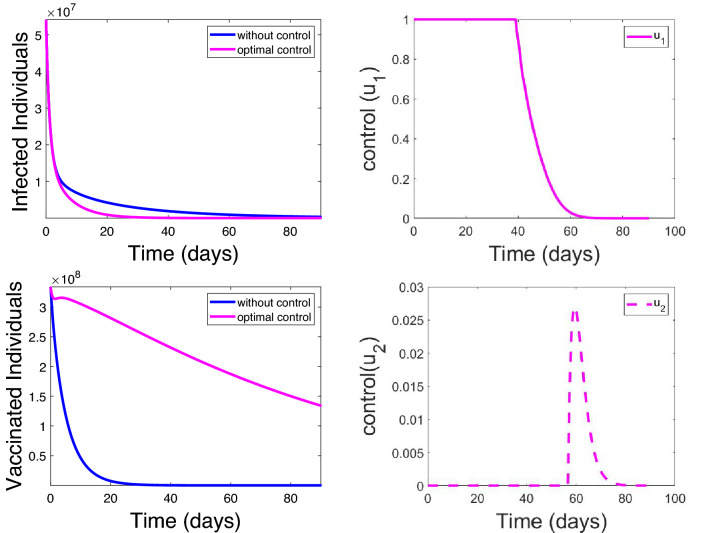
Effects of control strategies on the optimal control COVID-19 model

Figure 17 shows that our model fitted well with the selected countries data (daily cumulative number of reported cases).

**Fig. 18:**
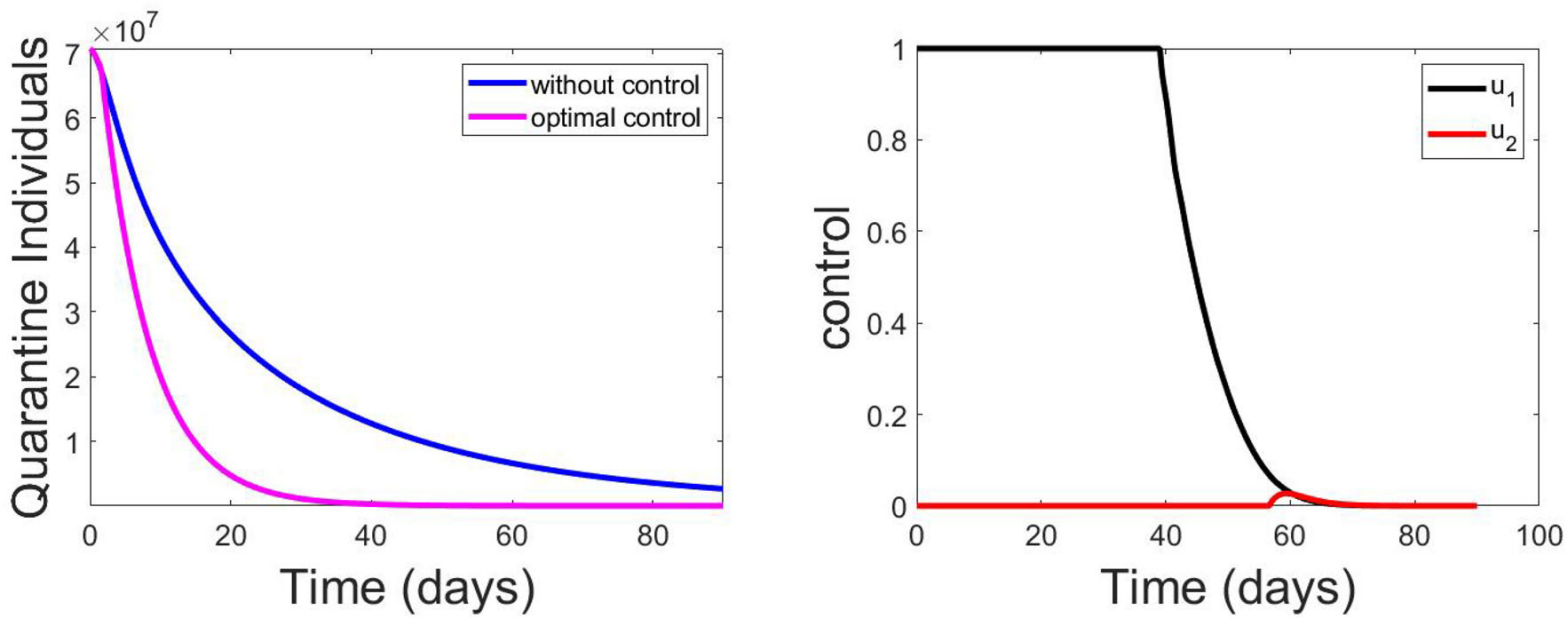
Effects of control strategies on the optimal control COVID-19 model

## Discussion

We used selected countries, confirmed cumulative cases in time series for the mortality data, recovered and infected cases obtained from the 22nd of January to the 20th of December 2021 by the Center for Systems Science and Engineering at Johns Hopkins University (2021) [54], to standardize the initial conditions of the model, the infective contact rate, $\beta$, and other parameter values in Table 2 considered as an explicit time function. Parameter fitting was established for the least squares nonlinear algorithm implementation in R software.

In this study, Fig. 3 depicts that compliance to social distancing in the USA drastically reduced the number of quarantined humans from February 20 to March 01, 2020 (days 30–40), when compared with the effect of non-compliance that occurred from January 22 to February 19, 2020 (day 1–29). The number of quarantined continued to decrease up until December 20, 2021, which is the 705 and last day of the analyzed data. This may be an indication of extensive efforts put in place by scholars to comprehend the bases of the virus and the adherent to the precautionary measures and guides put in place by decision makers. Figure 3 (lower left) also takes similar pattern with Fig. 3 (upper right), which indicates that the compliance of social distancing influences the number of infected humans after day 30. Our findings also showed, Fig. 3 (lower right), that the rate of social distancing compliance raises the recovery rate of infected humans in the USA. The analysis also revealed that the rate of human recovery started decreasing drastically after February 20, 2020 (after day 30), which may be as a result of reduction in compliance with social distancing. It is likely that the awareness of reduction in the number of quarantine led humans to relax in their observation of the social distance precaution. Also, this may be due to the development of another opportunistic illness from the virus.

We investigated the impact of social distancing on COVID-19 in Italy within January 22 to December 20, 2021 (day zero to 705), as shown in Fig. 4 (upper right and lower left); our results show that the implementation and compliance of social distance reduce the number of quarantines, and the rate of infected humans in Italy increases. This approach of social distancing also influenced the recovery rate of infected humans as shown in Fig. 4 (lower right), although with reduction in the recovery rate after day 30 of compliance. This may also be an indication of the people’s negligence to social distancing or increase in the number of opportunistic diseases caused by COVID-19 infection.

In South Africa, as depicted in our findings, Fig. 5 (upper right) shows that all the rate of social distancing compliance totally decreased infected quarantined individual numbers. Also the number of infected humans totally reduced as shown in Fig. 5 (lower left). The findings depicted that the compliance of the people to social distancing hampered and decreased the number of both the quarantined and infected humans. And as shown in Fig. 5 (lower right), social distancing also influenced the rate of human recovery from COVID-19, but the reduction in the compliance and adherence to the social distancing approach in South Africa gradually reduced the recovery rate.

In Nigeria, the influence of social distancing was observed in the number of quarantined and infected humans, as shown in Fig. 6 (upper right and lower left), respectively. It was discovered that compliance with social distancing approach declined the number of quarantined and COVID-19-infected humans, which showed that the approach really worked in containing the virus. On the number of recovered humans, it was observed, Fig. 6 (lower right), that compliance with social distancing regulation initially increased, but later decreased from February 20, 2020 (after day 30), which may be as a result of reduction in adherence to the regulation.

Figure 15 presents that the model is well fitted with the selected countries data (cumulative daily reported). Figure 17 (uppermost left) depicts that daily reported cases of COVID-19-infected individuals decrease but may not be eradicated without control, but Fig. 17 (uppermost right) shows that it may be eradicated with optimal control of both physical and vaccination. The blue line approaches the horizontal axis but does not touch it (asymptotic), but the pink line touched the horizontal line after some days. This shows that with optimal control the infected individuals can decrease faster to zero as time increases. Figure 17 (lower left) also depicts that vaccinated individuals decrease fast with time without control. However, Fig. 17 (lower right) shows that with optimal control of vaccination consumption, the vaccinated individual decreases slowly (lower right). This implies that when optimal control is enforced, vaccinated individuals recorded per day are more than when it is without control. Figure 18 (left) shows that decrease quarantine individuals decreases slowly without control, but Fig. 18 (right) shows that when optimal control of social distance and vaccination is enforced, quarantine individuals decrease faster with time. It should be noted that infected individuals and quarantine individuals decrease with time with the level of improvement in medical facilities, but it will decrease faster with optimal control. On the other hand, individuals vaccinated are expected to increase and then begin to decrease slowly when optimal control is enforced, but if optimal control is not enforced, the number of individuals vaccinated will decrease fast. People are not willing to be vaccinated, but with optimal control, more people will be vaccinated.

## Conclusion

The entire world is recently faced with devastating novel pandemic COVID-19 (coronavirus) that appeared in Wuhan, China, in December 2019. The fatal COVID-19 pandemic spreads to over 215 countries, with over 5.5 million confirmed cases and 347,379 total global deaths, and over 2.3 million recovery recorded so far. There is no effective or secure vaccine against COVID-19. Also, there are no protected and cogent antiviral drugs. Futhermore, to curb and mitigate against COVID-19, there are total compliance measures, such as physical distancing (lockdown of cities, closure of worship places, schools, malls, and other public gathering), isolation of suspected or confirmed cases, contact tracing, quarantining of established cases, and the public use of face masks. In this article, a new mathematical model formulated for the analysis with numerical simulation for a better understanding of the transmission dynamics and COVID-19 control in selected countries.

Generally, the present study formulated a model by paramet rizing COVID-19 data for the selected countries and their populations. The model was used to evaluate the influence of compliance (physical distancing) as intervention strategy. Some fundamental findings of the study are:

- This work is on physical distancing and other control policies as a means of non-pharmaceutical and also included pharmaceutical intervention. Our model is designed to prevent re-occurrence of different variants of the COVID-19 pandemic even if there are known vaccines.
- In this research, we have used statistical methods in estimating parameters of a mathematical dynamic model to adequately depict the real system under study, rather than just assuming values for the parameters, which might not depict the real system under study.
- We have also shown how a parameter, which is the rate of compliance with social distance practice, can be used to flatten the curve in real situations.
- In some countries of the world, the fear of contracting COVID-19 is now reducing because the curve is becoming flat, while the fear of collapsing the economy that has been built over the years is now increasing. Trying to balance not being infected with the virus and at the same time helping the poor state of the economy that is already threatened by the coronavirus is the aim of any responsible government. Therefore, it is necessary that the curve is flattened and that economies re-open as soon as possible.
- One of the ways to flatten the curve is through strict compliance with social (physical) distancing policy. The more the compliance level, the earlier the curve is flattened and the earlier the economy is re-opened.
- This research has used empirical results (real data) to show how compliance with social (physical) distancing can help in flattening the curve.
- We, hence, recommend strict social (physical) distancing policy by the government of countries already affected by the virus to quickly flatten the curve for safe re-opening of the economy in the nearest future
- Even if the economy is re-opened in the countries where the curves are already flattening, it is recommended that they still maintain the recommended physical distance of 1 meter, so that a second phase of the pandemic will not reoccur.

## Appendix

### Model parameter estimation

The model parameters are estimated from the real-time series and demographic data of the selected countries, using some simple estimation formula and least square estimation (LSE) techniques.

The natural death rate, $\mu$, is estimated by

$$\begin{aligned} \hat{\mu } =\frac{1}{\mu _{0}}; \mu _{0}>0 \end{aligned}$$

where $\mu _{0}$ is the life expectancy of a country before the breakout of COVID-19 gotten from demographic data of individual countries.

Equation (2.5) can be written such that COVID-19-induced death rate $\sigma$ can be estimated from it as

$$\begin{aligned} \hat{\sigma }\sum _{t=1}^{T}I_{t} = \sum _{t=1}^{T}D_{t}^{'} \end{aligned}$$

where $D_{t}^{'}$ is the change in COVID-19-induced death reported per day by Center for Disease Control (CDC) in the country, $I_{t}$ is the confirmed laboratory reported cases per day (infected compartment), and *t*, $(t = 1, 2,...,T)$ represents time measured in days, $T = 705$ is the number of days covered in the study, from 22nd January 2020 to 20th December 2021. This shows that out of infected individuals, how many individuals died of COVID-19. This rate will be zero at disease-free, but when there is disease, this rate increases as the number of death increases. It is always less that 1, since $I>D$. It is a fraction of infected individuals that are death.

The recovery rate $\gamma$ for infectious individuals (I) to recovery class (R) is given by

$$\begin{aligned} \hat{\gamma }\sum _{t=1}^{T}R_{t} = \sum _{t=1}^{T}I_{t} \end{aligned}$$

where $R_{t}$ is the reported recovered individuals from COVID-19 per day. The recovery rate $\gamma$ is a percentage of infected compartment who recovered.

From Eq. (2.3) at steady state, it is easy to see that the quarantine compartment is directly proportional to the infected compartment with *k* constant of proportionality, such that

$$\begin{aligned} \hat{k} =\frac{\hat{\mu }+\hat{\sigma }+\hat{\gamma }}{\hat{\theta }}; \hat{\theta }>0 \end{aligned}$$

The value of *k* can easily be gotten from data using the relationship $Q_{t}=\hat{k}I_{t}$ by LSE, and the value of $\theta$ can be estimated easily.

Rate at which recovered individuals (R) losses immunity and progress to susceptible class (S) denoted by $\delta _{1}$ is estimated by

$$\begin{aligned} \hat{\delta }_{1}\sum _{t=1}^{T}S_{t} = \sum _{t=1}^{T}R_{t} \end{aligned}$$

It should be noted that rate $\hat{\delta }_{1}$ is always less than $\hat{\gamma }$ at any time *t* since $S_{t}>I_{t}$.

At steady state, Eq. (2.4) can be written such that $\tau$ can be derived from it as

$$\begin{aligned} \hat{\tau } \sum _{t=1}^{T} S_{t}=(\hat{\mu }+\hat{\delta _{1}})\sum _{t=1}^{T}R_{t} - \hat{\gamma } \sum _{t=1}^{T}I_{t} \end{aligned}$$

At steady state, Eq. (2.2) can be written as

$$\begin{aligned} \sum _{t=1}^{T} S_{t}=\frac{[\hat{\mu }+\hat{\theta }+\delta _{0}(1-p_{1})]}{\lambda }\sum _{t=1}^{T}Q_{t} \end{aligned}$$

Every other parameters are estimated from the data, following the relationship between the variables at steady state.

Real-time series data collected on a daily basis (equal time sequence) for some days are used for estimating the parameters of the dynamic COVID-19 mathematical model using statistical estimation techniques. The parameters are estimated from data using least square techniques. The 95% confidence interval (CI) of each parameter is fitted using the parameter estimate plus or minus an error bound. The error bound is 1.96 times the standard error of estimate. Some of the parameters are estimated using known static formulas, while some are fitted using stochastic models. Some important parameters are varied within its possible values and some within their confidence limits in the dynamic modeling, such as rate of compliance with social distance practice. This is possible, since all the parameters and variables are related in one way or the other, either linearly or nonlinearly. The real data used, which are known variables, are data on laboratory-confirmed COVID-19 reported cases, *I*(*t*) for the selected countries, recovered cases *R*(*t*) and total deaths *D*(*t*). The quarantine class, *Q*(*t*), are estimated from the model based on their relationship with known variables (*I*(*t*), *R*(*t*) *and* *D*(*t*)). The susceptible class, *S*(*t*), are estimated from the country’s population, *N*(*t*) and the relationship with known variables (*I*(*t*), *R*(*t*) *and* *D*(*t*)). It is assumed that all individuals in a country selected are susceptible, if they are not infected. So that $N(t)=S(t)+Q(t)+I(t)+R(t)+D(t)$. This relationship made it possible to estimate the susceptible class.

**Table 4:** COVID-19 active, recovered, death, and cum. infected cases

Countries	Active	Recovered	Death	Cum. infected
US	669,903	72,329	42,094	784,326
Spain	98,771	80,587	20,852	200,210
Italy	108,237	48,877	24,114	181,228
France	97,601	37,409	20,265	155,275
Germany	50,703	91,500	4,862	147,065
Turkey	75,410	13,430	2,140	90,980
China	1,436	77,745	4,636	83,817
Iran	19,023	59,273	5,209	83,505
Brazil	16,026	22,130	2,587	40,743
Belgium	25,260	8,895	5,828	39,983
Canada	23,,373	12,543	1,726	37,642
Switzerland	7,915	18,600	1,429	27,944
South Africa	2,187	1,055	58	3,300
Nigeria	455	188	22	665
Mean	85,450	38,897	9,702	134,049
Standard Dev.	172,688	32,244	12,415	198,069
Maximum	669,903	91,500	42,094	784,326
Minimum	455	188	22	665

## COVID-19 exploratory data analysis of selected countries

In statistics, exploratory data analysis (EDA) is an approach to analyzing data sets to summarize their main characteristics, often with visual methods [53, 55]. A statistical model can be used or not, but primarily EDA is for seeing the story the data can tell us beyond the inferences (modeling or hypothesis testing). EDA will expose the hidden features inherent in a data dataset [56, 57]. [58, 59] promoted EDA and mentioned that statisticians have placed too much emphasis on inference (statistical hypothesis testing, that is, confirmatory data analysis), so, more emphasis needed to be placed on using data to suggest hypotheses to test. In particular, he held that confusing the two types of analyses and employing them on the same set of data can lead to systematic bias owing to the issues inherent in testing hypotheses suggested by the data. So, EDA would help to identify the best model to be fitted and the best hypothesis to be tested.

In this research, four countries are selected from 15 countries for study. These countries are USA, Italy, South African, and Nigeria. The data collected on these countries are compared with that of China using different EDA plots such as multiple time plot, bar plots, histogram with kernel density curves, box plots, QQ plots, and scatter plots. These plots will help tell the story behind each variable considered for the countries under study. Actual values are used in most cases while in few occasion log of the actual values were taken for better comparison.

Table 4 shows that as at April 20, 2020, USA has the highest COVID-19-confirmed laboratory cases (cum. infected) among the selected countries of the world, followed by Spain then Italy. The figure of USA and Spain alone are more than the total of the rest 13 countries under study. USA has the highest active cases followed by Italy, then Spain. Germany, Spain, and China in that order have the highest recovered cases among the selected countries as at the time of this report. USA, Italy, Spain in that order recorded the highest COVID-19-induced deaths among the selected countries for the period under review. The standard deviation and range (maximum - minimum) show that the incident of COVID-19 is very high in some countries and very low in some other countries making the variability to be very high. As at the time of this report, South Africa and Nigeria both in Africa have the lowest confirmed cases of 3,300 and 665, respectively. All the countries selected outside Africa have well over 25,000 confirmed cases in the first 705 days of reporting the virus.
